# Autologous human iPSC–derived alveolus-on-chip reveals early pathological events of *Mycobacterium tuberculosis* infection

**DOI:** 10.1126/sciadv.aea9874

**Published:** 2026-01-01

**Authors:** Chak Hon Luk, Gabriel L. Conway, Kim Jee Goh, Antony Fearns, Irene Rodriguez Hernandez, Nathan J. Day, Natalia Athanasiadi, Rocco D’Antuono, Enrica Pellegrino, Janick D. Stucki, Nina Hobi, Maximiliano G. Gutierrez

**Affiliations:** ^1^ Host-Pathogen Interactions in Tuberculosis Laboratory, The Francis Crick Institute, London, UK.; ^2^ Bioinformatics and Biostatistics Science and Technology Platform, The Francis Crick Institute, London NW1 1AT, UK.; ^3^ Crick Advanced Light Microscopy STP, The Francis Crick Institute, 1 Midland Road, NW1 1AT, London, UK.; ^4^ Department of Biomedical Engineering, School of Biological Sciences, University of Reading, Reading, UK.; ^5^ Alveolix AG, Swiss Organs-on-Chip Innovation, Bern, Switzerland.

## Abstract

Immunocompetent and experimentally accessible alveolar systems to study human respiratory diseases are lacking. Here, we developed a single-donor human induced pluripotent stem cell-derived lung-on-chip (iLoC) containing type II and I alveolar epithelial cells, vascular endothelial cells, and macrophages in a microfluidic device that mimic lung three-dimensional mechanical stretching and air-liquid interface. Imaging and single-cell RNA sequencing analysis revealed that the iLoC recapitulated cellular profiles present in the human distal lung. Infection of the iLoC with the human pathogen *Mycobacterium tuberculosis* (Mtb) showed that both macrophages and epithelial cells were infected but not permissive to bacterial replication. Stochastically, large macrophage clusters containing necrotic macrophages supporting Mtb replication were observed. A genetically engineered autophagy-deficient iLoC revealed that after Mtb infection, macrophage necrosis was higher upon ATG14 deficiency without bacterial replication. Together, we report an autologous, genetically tractable human alveolar model to study lung diseases and therapies.

## Teaser

An autologous, genetically tractable human iPSC–derived alveolar model is developed to study tuberculosis.

## INTRODUCTION

The human alveolus is an important tissue microenvironment for material exchange while being safeguarded by tissue-resident immune cells against respiratory pathogens, including coronaviruses and *Mycobacterium tuberculosis* (Mtb) (*1*). Given its significance in homeostasis (*2*) and promise for drug delivery, many in vitro human models have been developed to circumvent the differences in anatomy, immune cell composition, and disease pathogenesis between human and animals (*3*). Motivated by the FDA Modernization Act 2.0 (*4*), organ-on-chip (OoC) technologies emerge as predictive tissue modeling tools and reliable alternative to animal testing backed by hypothesis-based and unbiased studies (*5*). In the past decade, numerous efforts aimed to recreate pulmonary microenvironment on microfluidic devices (e.g., chips), where these systems can be categorized by their choices of organism (i.e., human or murine), cell source (i.e., cell line, primary cells, or stem cell derived), and target tissue (nasal, airway, or alveolus). There are several reported multicellular airway-on-chips (*6*, *7*) and alveolus-on-chips (*8*–*17*) that are based on cell lines (*14*) and primary cells (*6*–*12*, *15*–*19*) or a combination of both (*13*, *20*). A major challenge in building robust and scalable distal lung models is the access to a virtually unlimited source of reliable, standardized, phenotypically characterized, and immunocompetent alveolar cells from a tractable single donor. Despite that the use of human induced pluripotent stem cell (iPSC) technologies satisfied most of these requirements and successful examples of developing alveolar epithelial cells (*21*–*29*), work combining both OoC and iPSC to mimic the distal lung is limited.

Here, we report the engineering of a physiologically inspired human lung-on-chip that combines human iPSC with lung-on-chip technologies. We built an autologous, experimentally accessible, multicellular lung-onchip from a single iPSC source that we named iLoC (for iPSC-derived lung-on-chip). The iLoC enables the incorporation of genetically engineered cells with an isogenic background. Using single-cell transcriptomics, we demonstrate an important role of endothelial cells in regulating tissue innate and adaptive immune responses. Applying the iLoC as an early infection model for tuberculosis (TB), we found that both epithelial cells and macrophages are infected with Mtb, and cell death predominantly takes place in macrophages. Last, by combining the iLoC with genetically engineered iPSC, we found a cell type-dependent role for the autophagy gene *ATG14* in regulating macrophage survival during Mtb infection.

## RESULTS

### Lung progenitors differentiate into iAT2 and iAT1 cells in a lung-on-chip microfluidic device

**Fig. 1.**
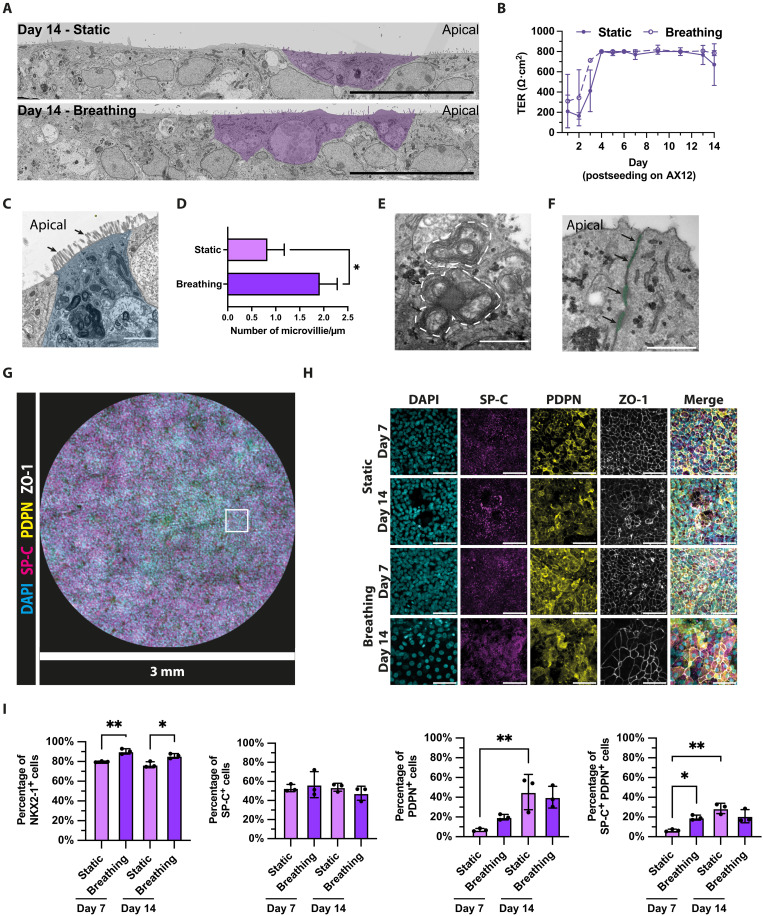
iPSC-derived iAT2 and iAT1s are differentiated in a lung-on-chip microfluidic device. (**A**) Representative TEM images of iAT2 and iAT1 on AX12 at 14 days postseeding under static or breathing conditions. A fraction of the iAT2 with microvilli are highlighted in light purple. Scale bar, 20 μm. (**B**) TER quantification of iAE on AX12 up to 14 days postseeding, under static (solid line) or breathing (dotted line) conditions; means ± SD, *n* = 18 technical replicates from *n* = 3 independent experiments. (**C**) TEM image showing a magnified view of microvilli on putative iAT2 cells (highlighted in blue) differentiated in AX12 at 21 days postseeding, cilia are indicated with arrows. Scale bar, 2 μm. (**D**) Quantification of microvilli ultrastructure in (A), *n* > 30 quantified fields of view (FOVs) from three independent iAE chips. (**E**) TEM image showing LB-like structures in putative iAT2 cells differentiated in AX12 at 10 days postseeding, LBs are highlighted by dotted lines. Scale bar, 500 nm. (**F**) TEM image showing a magnified view of desmosomes in iAE differentiated in AX12 at 21 days postseeding; desmosomes are highlighted in green and indicated with arrows. Scale bar, 500 nm. (**G**) Representative image of the entire AX12 membrane with iAT2 and iAT1 cells showing nuclei (cyan), SP-C (magenta), PDPN (yellow), and ZO-1 tight junctions (white) at day 7 postseeding. Scale bar, 3 mm. (**H**) Representative zoomed images of iAT2 and iAT1 differentiated on AX12 showing nuclei (cyan), SP-C (magenta), PDPN (yellow), and ZO-1 tight junctions (white) at day 7 and day 14 postseeding, under static or breathing conditions. Equivalent area as depicted by square in (G). Scale bar, 50 μm. (**I**) Quantification of NKX2-1, SP-C, and PDPN-expressing cells in iAE at day 7 and day 14 postseeding, under static or breathing conditions; means ± SD, *n* = 3 independent experiments, one-way analysis of variance (ANOVA). *P* value: *P < 0.05, **P < 0.01.

To generate iPSC-derived type I and type II alveolar epithelial cells (iAT1 and iAT2, respectively) as well as a biological barrier, we modified previously described protocols (*23*, *25*). We differentiated iAT2 from human iPSC, passing through definitive endoderm, anterior foregut endoderm, and ventralized anterior foregut endoderm stages into lung progenitors (iLungPro). The iLungPro were enriched using a carboxypeptidase M (CPM)^hi^ or CPM^+^ surrogate enrichment strategy (fig. S1A). This optimized protocol rendered the highest abundance of CPM^+^ iLungPro and required ventralization of anterior foregut endoderm compared to prior protocols (fig. S1, B to F) (*23*, *25*). We adopted the ^AX^Lung-on-Chip System, consisting of two electropneumatic devices, ^AX^Exchanger and ^AX^Breather, that interfaces with the OoC device (AX12) to provide a physiological three-dimensional (3D) stretching of the biocompatible silicon membrane as well as other maintenance function (fig. S1G) (*8*). The enriched iLungPro were seeded directly on the Matrigel-coated AX12 to establish an epithelial layer both under static and breathing conditions (Fig. 1A), where mechanical stretch is introduced 4 days postseeding as a strong transbarrier electrical resistance (TER) developed (Fig. 1B). Recent work induced iAT2-iAT1 differentiation by pharmacological activation of YAP/TAZ pathway (*21*, *29*). Herein, we initiated air-liquid interface (ALI) at 7 days postseeding to induce iAT2-iAT1 differentiation as it offers an accessible apical epithelium and minimizes medium composition change that is crucial to coculture at later steps. The enrichment of CPM^+^ iLungPro was critical for barrier function (fig. S1H), and the TER was maintained in both static and breathing conditions, indicating that the epithelial barrier remained functional up to 14 days (Fig. 1B).

Using scanning electron microscopy (SEM), we observed the presence of microvilli structures covering the surface of the epithelium that prominently appeared at day 14 postseeding (fig. S2A). Visualizing the microvilli structures using transmission electron microscopy (TEM) (Fig. 1, A and C), we observed an enrichment of microvilli in the presence of mechanical stretch (Fig. 1D). At the ultrastructural level, TEM analysis showed that a substantial proportion of the cells exhibited typical microvilli features of AT2 cells and some of these cells displayed a cuboid morphology (Fig. 1C) (*30*). Using fluorescence microscopy, we demonstrated the absence of tubulin-based ciliated structures at the apical side of iAT2, indicating the identity as microvilli (fig. S2B). Notably, we observed the presence of lamellar body (LB)-like ultrastructure with typically lamella structure and semidense core, organelles specific for storage and secretion of surfactants, in iAT2 cells (Fig. 1E and fig. S2C) (*31*). At the ultrastructural level, we also observed the presence of a functional barrier with tight junctions and desmosomes (Fig. 1B) as previously described in human lungs (*32*). At day 14 postseeding, we observed regions of different thicknesses contributed by the multilayers of cells, indicating that the iPSC-derived alveolar epithelium (iAE) remains proliferative and harbor an intrinsic self-organization program. Glycogen lakes serve as cellular site for surfactant synthesis and LB formation (*33*, *34*). The observation of abundant glycogen lakes suggests active production of surfactant by the iAE (fig. S2D).

We phenotypically profiled the iAT2 and iAT1 differentiated on AX12 by single-cell imaging analysis (Fig. 1G) and observed the expression of mature surfactant C (SP-C, iAT2 marker) and podoplanin (PDPN, iAT1 marker) in differentiated iAT2 and iAT1 (Fig. 1H). At days 7 and 14, most of the cells expressed the marker of distal lung epithelium NKX2-1 at high levels, where breathing motion achieved a higher number of NKX2-1^+^ cells (Fig. 1I and fig. S3A). Comparing the static and breathing conditions, we observed a comparable abundance of mature SP-C^+^ cells in both conditions at days 7 and 14, while a higher fraction of PDPN^+^ cells is observed under breathing conditions at day 7 and both increased to a comparable fraction at day 14 (Fig. 1, H and I). With dual labeling of mature SP-C and PDPN, we observed SP-C/PDPN double-positive cells at days 7 and 14, indicating the presence of intermediate cell states. Notably, we also observed distinct morphologies of mature SP-C^+^ and PDPN^+^ cells (Fig. 1H and fig. S3B). We further validated the expression of iAT2 and iAT1 marker genes in the differentiated iAE and benchmarked to primary human adult lung samples. The levels of expression of *NKX2-1* (alveolar epithelial marker), *SFTPC*, *SLC34A2* (AT2 markers), *PDPN*, and *CAV1* (AT1 markers) increased as iLungPro differentiated into iAT2 and iAT1, yet the overall levels were lower than that in human lung (fig. S3C). Together, we concluded that iAT2 and iAT1 differentiated on AX12 formed a functional epithelial barrier showing typical features of the alveolar epithelium observed in the adult human lung that includes the formation of LBs, cilia, tight junctions, and desmosomes.

### The microfluidic device enabled the formation of a functional epithelial-endothelial barrier

**Fig. 2.**
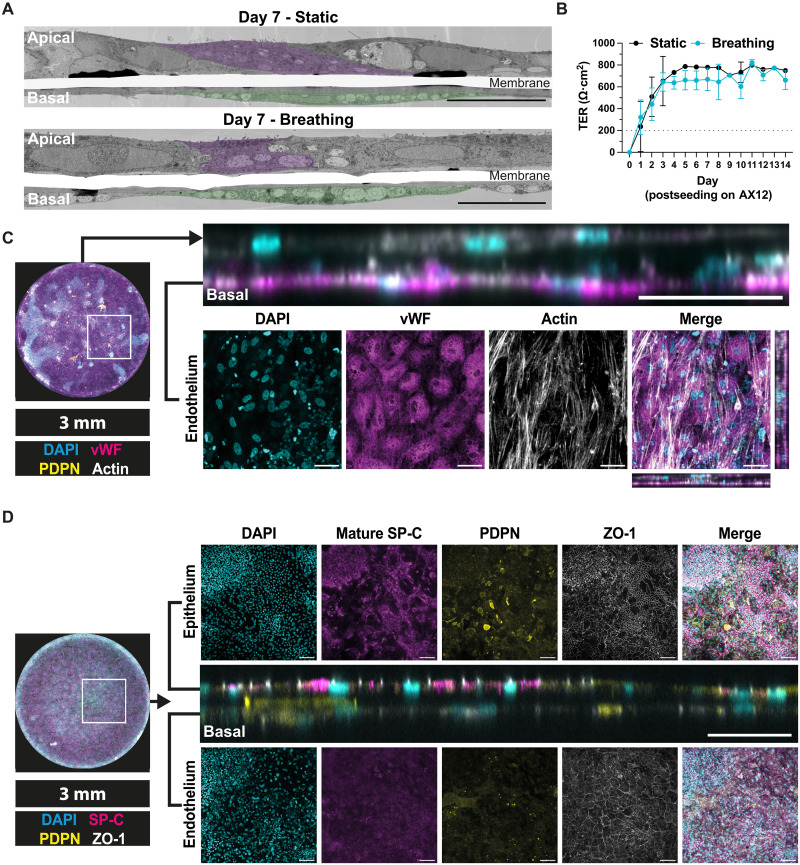
iPSC-derived iVECs with iAE barrier build a dual epithelium/endothelium in a lung-on-chip microfluidic device. (**A**) Representative TEM images of iAT2 and iVEC on AX12 at 7 days postseeding, under static or breathing conditions, representative iAT2 and iVEC are highlighted in purple and green, respectively. Scale bar, 20 μm. (**B**) TER measurement of iLoC up to 13 days post-iLoC assembly under static (black) or breathing (cyan) conditions; means ± SD, *n* = 18 technical replicates from *n* = 3 independent experiments. (**C**) Representative images of orthogonal and planar views of iVEC cultured on basal side of AX12 together with apical iAT2 and iAT1 cells at day 15 post-iLoC assembly, images showing nuclei (cyan), vWF (magenta), and actin (white). Scale bar, 50 μm. (**D**) Representative images and orthogonal and planar views of iVEC cultured on basal side of AX12 together with apical iAT2 and iAT1 cells at day 15 post-iLoC assembly, images showing maximum projection of epithelium and endothelium, nuclei (cyan), mature SP-C (magenta), PDPN (yellow), and ZO-1 (white). Scale bar, 50 μm (orthogonal view) and 100 μm (planar view).

To differentiate vascular endothelial cells (iVECs) from human iPSC for the basal component of iLoC, we adopted a previously described protocol (*35*). We validated the expression of the endothelial marker CD31 and the vascular endothelial marker CD144 by fluorescence microscopy (fig. S4A). Cell surface expression of CD31, CD34, CD144, and KDR was also analyzed by flow cytometry (fig. S4B) as well as *CD31*, *CD34*, *CD144*, and intracellular marker von Willebrand factor (*vWF*) by reverse transcription quantitative polymerase chain reaction (RT-qPCR) against human umbilical vein endothelial cells (fig. S4C). To confirm the cellular function of iVEC, we evaluated the capacity of very low-density lipoprotein uptake and tubules formation in the angiogenesis assay (fig. S4, D and E). The differentiated iVECs were seeded onto the basal side of the AX12, and iLungPro were subsequently seeded onto the apical side 1 to 2 days after the seeding of iVEC. The three cell types (iAT2, iAT1, and iVEC) together formed an epithelium-endothelium bilayer that maintained an ALI and TER up to 14 days under both static and breathing conditions initiated at the same time as iAE-only cultures (Fig. 2, A and B). The iAE and iVEC remained as intact bilayers, and basal iVEC displayed a sustained vWF expression and a typical arrangement of actin cytoskeleton after 15 days of coculture (Fig. 2C). The patterned expression of mature SP-C and PDPN was maintained in the iAE despite different cell morphology to that in the absence of iVEC, and ZO-1 tight junctions were present in both iAE and iVEC endothelium (Figs. 1H and 2D). We concluded that the three cell types were maintained in the AX12 as an epithelial-endothelial functional barrier and preserving both epithelial and endothelial cellular phenotypes for at least 14 days.

### Functional macrophages integrated into the epithelial barrier of the microfluidic device

**Fig. 3.**
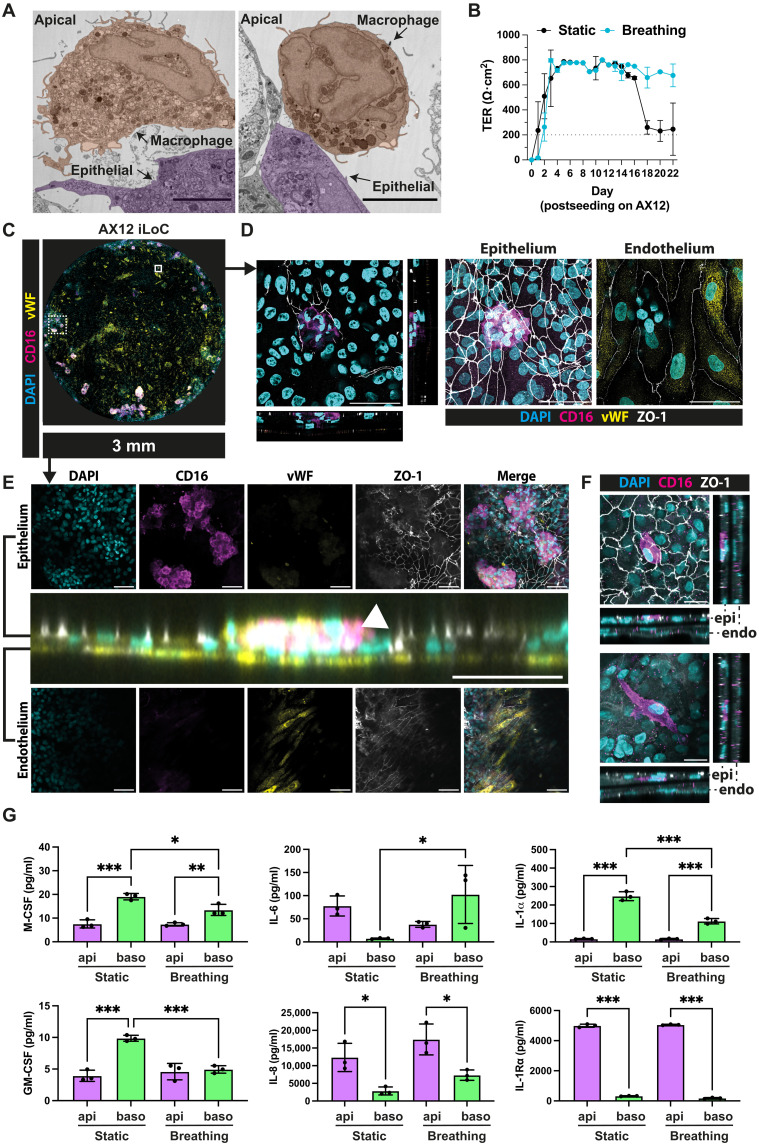
Incorporation of iPSDM with iAE-iVEC barrier to form an immunocompetent alveolar microenvironment in a lung-on-chip microfluidic device. (**A**) Representative TEM images of iLoC at day 15 post-iLoC assembly without mechanical stretch; zoom in shows iPSDM resting on top of iAE. Representative iAE and iPSDM are highlighted in purple and orange, respectively. Scale bar, 5 μm. (**B**) TER measurement of iLoC up to 22 days post-iLoC assembly under static (black) or breathing (cyan) conditions; means ± SD, *n* = 18 technical replicates from *n* = 3 independent experiments. (**C**) Representative images of an overview of AX12 iLoC images showing nuclei (cyan), CD16 (magenta), vWF (yellow), and ZO-1 (white). Scale bar, 3 mm. (**D**) Representative confocal images depicted by solid line square in (C); orthogonal and planar views of iPSDM residing on alveolar epithelium and maximum projection of epithelium and endothelium of AX12 iLoC, at day 21 post-iLoC assembly; images showing nuclei (cyan), CD16 (magenta), vWF (yellow), and ZO-1 (white). Scale bar, 50 μm. (**E**) Representative confocal images depicted by dotted line square in (C); orthogonal and planar views of iLoC epithelium and endothelium with iPSDM (arrowhead) residing on alveolar epithelium; images showing nuclei (cyan), CD16 (magenta), vWF (yellow), and ZO-1 (white). Scale bar, 50 μm (orthogonal view) and 100 μm (planar view). (**F**) Representative images of planar and orthogonal views of iPSDM residing on top of (top) and integrated beneath (bottom) alveolar epithelium at day 21 post-iLoC assembly, images showing nuclei (cyan), CD16 (magenta), and ZO-1 (white). Scale bar, 20 μm. (**G**) Quantification of M-CSF, GM-CSF, IL-6, IL-8, IL-1α, and IL-1Rα secretion from the epithelial and endothelial side of iLoC under static and breathing conditions; means ± SD, *n* = 3 independent experiments, one-way ANOVA. *P* value: *P < 0.05, **P < 0.01, *** P < 0.001.

Human alveoli contain alveolar macrophages essential for tissue homeostasis and immune surveillance, inspiring us to incorporate iPSC-derived macrophages (iPSDM) into the iAE-iVEC coculture. We used a well-characterized protocol for monocytes and macrophages (*36*), where iPSC-derived monocytes (iPSDMono) are differentiated with granulocyte-macrophage colony-stimulating factor (GM-CSF) to yield iPSDM of an alveolar-like phenotype (*37*, *38*) that is also known as an M1/M2 hybrid phenotype in human macrophages (*39*). iPSDM analyzed by flow cytometry and fluorescence microscopy were positive for CD11b, CD16, CD14, CD68, CD169, CD64, and MARCO and negative for CD163 and HLA-DR (fig. S5, A and B). Differentiated iPSDM were seeded to achieve an optimal ratio of 1:10 to 1:20 (macrophage/epithelial cells) in the iLoC to represent that in human alveolar space (*40*). At the ultrastructural level, iPSDM were loosely associated with the epithelium and displayed intracellular vacuoles (Fig. 3A). With the presence of iPSDM, the barrier function of the iLoC remained intact (≥200 Ω·cm^2^), and the breathing of the iLoC achieved a more stabilized barrier function after 15 days of seeding (Fig. 3B). The TER in breathing condition was closer to physiological relevant ranges that is likely contributed by the breathing motions (initiated on day 4 of iLungPro seeding) in mimicking alveolar physiology (Fig. 3B). Notably, we found that iPSDM occasionally form clusters that associated with discrete regions on the iAE, and the association of macrophages induced an invagination of the epithelial barrier (Fig. 3, C to E, and fig. S6, A and B). We confirmed that the ZO-1 tight junctions were intact on both the epithelium and endothelium, and iPSDM and iVEC were CD16 and vWF positive, respectively (Fig. 3, D and E, and fig. S7A). Furthermore, iAT2 (SP-C and ABCA3), iAT1 (AGER), and iVEC (CD31 and CD144) markers were present among the iAE and iVEC in iLoC, respectively, where SP-C was also detected in iPSDM, demonstrating the uptake of surfactant proteins, a key homeostatic function of alveolar macrophages (figs. S8, A to D, and S9A) (*41*). The iPSDM were dwelling in different niches in the iLoC, either residing on the epithelium or integrated beneath the epithelium (Fig. 3F). When we measured the secretion of cytokines, we observed the secretion of cytokines important for macrophage differentiation and homeostasis (M-CSF and GM-CSF) (*42*) as well as pro- and anti-inflammatory cytokines [interleukin-6 (IL-6), IL-8, IL-1α, and IL-1Rα] (Fig. 3G) (*43*–*45*). For the macrophage homing factors (M-CSF and GM-CSF), we observed higher levels of secretion at the basolateral side under static conditions, while such distinctions were less evident under breathing conditions. For the pro- and anti-inflammatory cytokines, we observed polarized secretion of IL-8, IL-1α, and IL-1Rα under both static and breathing conditions, where antagonistic immunoregulators (IL-1α and IL-1Rα) were enriched in opposite compartments of iLoC, further demonstrating the iLoC as a functionally organized epithelial-endothelial barrier. When comparing static and breathing conditions, we observed distinct secretion of M-CSF, IL-6, and IL-1α in the basolateral but not the apical compartment of the iLoC, suggesting a specific influence of breathing motion toward the endothelium (Fig. 3G). The lower level of M-CSF and GM-CSF detected in apical side can be accounted by the uptake by iPSDM for cellular homeostasis. Together, these data show that the iLoC is stable for at least 6 days with four cell types in the presence of both ALI and breathing.

### The iLoC benchmarks human alveolar lung cells

**Fig. 4.**
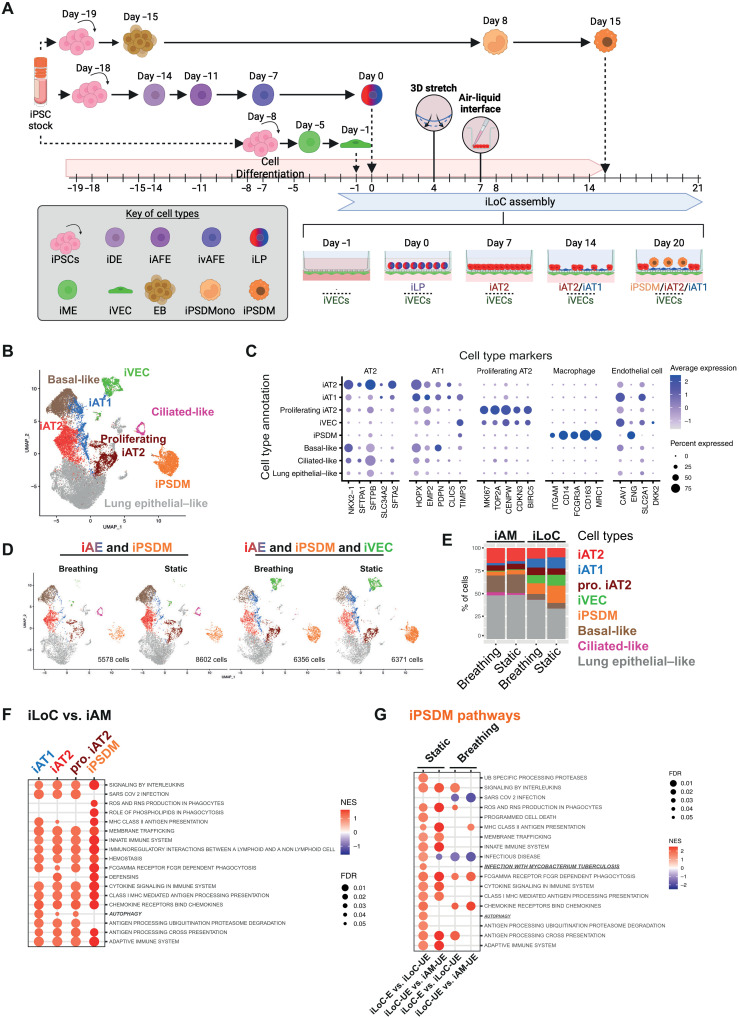
The four-cell type iLoC benchmarks human alveolar lung cells. (**A**) Schematic outline of iAT2, iAT1, iVEC, and iPSDM differentiation from iPSC and detailed steps of the iLoC assembly. (**B**) Uniform Manifold Approximation and Projection (UMAP) with cell annotations of an integrated analysis of 26,907 iAM or iLoC-derived cells in the presence or absence of mechanical stretch. (*n* = 1 per condition). (**C**) Dot plot representation of the expression of selected AT1, AT2, proliferating AT2, macrophage, and endothelial cell gene markers across annotated cell types. (**D**) UMAP visualization of the integrated iAM or iLoC individual samples in the presence or absence of mechanical stretch from (B) split by experimental condition. (**E**) Percentage of cells corresponding to the specific cell types (indicated with the color codes) in each individual sample from (B and D). (**F**) Dot plot showing selected Reactome enriched pathways from Gene Set Enrichment Analysis (GSEA) for differential expression analysis in iAT2, iAT1, proliferating iAT2, and iPSDM cell types under static condition in the presence or absence of iVEC. (iLoC versus iAM) (**G**) Dot plot showing selected Reactome enriched pathways from GSEA analysis for differential expression analysis between enriched and unenriched iPSDM subclusters in iLoC (iLoC-E versus iLoC-UE) and between unenriched iPSDM subclusters in iLoC and iAM (iLoC-UE versus iAM-UE) in presence or absence of iVEC and under breathing or static conditions. Created in BioRender. Luk, J. (2026) https://BioRender.com/wirsrvz.

The generation of the iLoC takes over 40 days from the iPSC stage, where macrophages are added and remain associated with the iLoC for at least 6 days (Fig. 4A). To define the cellular and phenotypic cell states on the iLoC, we profiled the iLoC using single-cell RNA sequencing (scRNA-seq). We focused on the significance of the endothelial barrier and breathing motion to the iLoC, represented by coculture of iAE-iPSDM (iAM) and iLoC under static or breathing conditions. Using a cellular marker classification approach, we identified the key cell types, iAT2, iAT1, proliferating iAT2 (pro. iAT2), iVEC, and iPSDM alongside basal-like, ciliated-like, and lung epithelial cells (Fig. 4, A and B, and fig. S10, A and B). Using the defined cell type annotation, we detected an elevated expression of cell type-specific marker genes or a combination of the marker genes that contributed to the cell type annotations and tissue specificity (Fig. 4C and figs. S10C and S11, A and B) (*21*, *46*). It is notable that the key iAT2 and iAT1 markers showed a lower expression than previously reported (*21*, *28*, *29*), yet we are able to detect several top ranked markers being reported (figs. S10C and S11, A and C) (*21*). Next, we mapped the sequenced cells onto a lung atlas with only datasets from healthy donors (fig. S12, A and B) (*46*). We observed that the iLoC-derived cells were mapped onto the clusters of epithelial cells, stromal cells, and macrophage/dendritic cells clusters (fig. S12B). Using clustifyr Spearman’s correlation to examine gene expression similarity between annotated cell types in the lung atlas and our cluster cells, our cells were predicted to recapitulate a continuum of cellular states of alveolar epithelial cells as well as alveolar macrophages (fig. S12C). Combining the cellular marker annotation and cell type prediction, this analysis showed the successful recreation of an alveolar microenvironment that mimic endogenous cell-cell communication and intermediate cellular states.

The presence of an epithelial-endothelial barrier association and mechanical stretch by breathing are believed to be important components of the latest generation of lung-on-chip (*5*). Thus, we experimentally analyzed the significance of breathing and presence of iVEC to iLoC using scRNA-seq. Comparing the four conditions, we observed a remarkable difference between the presence and absence of iVEC (iLoC versus iAM), where the presence of iVEC triggered a more stable iPSDM residence, iAT2-iAT1 transdifferentiation, and distal lung cellular identity (Fig. 4, D and E). Considering that the annotated basal-like cells and iAT1 both expressed *PDPN*, this could account for the distinct morphology of PDPN^+^ cells in Figs. 1H and 2D. On the contrary, breathing motion of the iLoC appears to have a less profound effect on the composition of cells (Fig. 4, D and E). To better understand the impact of iVEC and breathing motion on iLoC cellular functions, we performed Reactome pathway analysis. We observed a general enrichment of immune-related pathways in iAT2, iAT1, pro. iAT2, and iPSDM in the presence of iVEC (iLoC condition). The enriched pathways in iAT2, iAT1, and pro. iAT2 included antiviral and antibacterial pathways as well as oxidative phosphorylation, while iPSDMs were poised toward lysosomal degradation, phagocytosis, cytokine responses, and antigen presentation (Fig. 4F). Strikingly, the breathing motion induced a general down-regulation of immune-related pathways in iAT2, iAT1, pro iAT2, and iPSDM, and an up-regulation in iVEC, indicating a cell type-specific impact of breathing motion and a potential role of mechanical forces in maintaining a naïve immune state (fig. S13A and data S1). Together, these data show that the iLoC benchmarked key cellular components of human lung.

### Basal endothelial cells affect apical macrophage and epithelial phenotypes in the iLoC

Owing to the significance of immune cells in alveolar homeostasis, we investigated the iPSDM phenotypes in the iLoC. For this, iPSDM cells were extracted, reanalyzed, and clustered de novo. We observed that distinct subclusters uniquely enriched in the presence of iVEC (subclusters 2, 5, 6, and 8, which we named as iLoC-E) irrespective of the mechanical stretching, while the remaining are considered as unenriched clusters (subcluster 0, 1, 3, 4, 7, and 9, which we named iLoC-UE) (fig. S14, A to D). The subclusters of iPSDM showed a high expression of *CD86* and *MRC1* as well as *CD68*, *TLR2*, and *TLR4* (M1 markers) and *CD163*, *IL1R2*, and *IL10RA* (M2 markers). These findings are in agreement with previously reported M1/M2 phenotypes and cell type-specific markers of alveolar macrophages, suggesting that the iLoC microenvironment represents the alveolus for macrophage phenotype (fig. S14, E and F) (*39*, *47*). We observed that one of the iPSDM subclusters highly correlated to proliferating alveolar macrophages, while other subclusters closer represented alveolar or interstitial macrophages (fig. S14G). We further compared the iPSDM in the iLoC with the different subset of alveolar macrophages from bronchoalveolar lavage (*48*). We observed the expression of marker genes among subclusters of iPSDM (fig. S15A). The iPSDM in iLoC were predominately predicted to be alveolar macrophages with a minor fraction of alveolar/interstitial macrophage and negligible interstitial macrophages, indicating the capacity of iLoC to preserve alveolar macrophage identity (fig. S15, B and C). Comparing the iLoC-enriched iPSDM subclusters to the iLoC-unenriched subclusters (iLoC-E versus iLoC-UE), the iLoC-E macrophages displayed a higher expression in pathways of innate and adaptive immunity under both static and breathing conditions (Fig. 4G). At the same time, the iLoC-UE macrophages also up-regulated these immune pathways as compared to the counterparts in iAM samples (iAM-UE), suggesting that there are three populations of iPSDM with different immune function (Fig. 4G). As macrophage metabolism serves as a key determinant of macrophage phenotype and immune functions, we sought to further analyze the macrophage phenotypes and metabolic states (*49*). Using a recently reported scRNA-seq tool for monocyte/macrophage metabolic profiling (*50*). We found that the iPSDM in iLoC showed a “healing” phenotype, while macrophages appeared to shift from “transitional” to “inflammatory” states in the presence of iVEC, in agreement with the pathway analysis (fig. S16, A to C). Focusing on glycolysis, oxidative phosphorylation, and fatty acid metabolism, the iLoC-E iPSDM showed high expression levels of oxidative phosphorylation genes, while glycolysis and fatty acid metabolism were less distinctive among iPSDM subclusters (fig. S16D). We also labeled γ-aminobutyric acid (GABA), a key driver of macrophage metabolism, cytokine responses (*51*), and antimicrobial activity (*52*, *53*). Notably, we observed heterogenous levels of GABA among iPSDM in iLoC (fig. S17A). Together, these data show that iVEC not only supported the retention of macrophages and global up-regulation of immune pathways but also increased the fraction of macrophages that up-regulate key immune pathways. This indicates that coculturing of macrophages with relevant cells in the iLoC environment imprints macrophage phenotype and function. We concluded that the iLoC mimics the cellular diversity and heterogeneity of human distal lung tissues.

### The iLoC mimics early stages of infection with Mtb

**Fig. 5.**
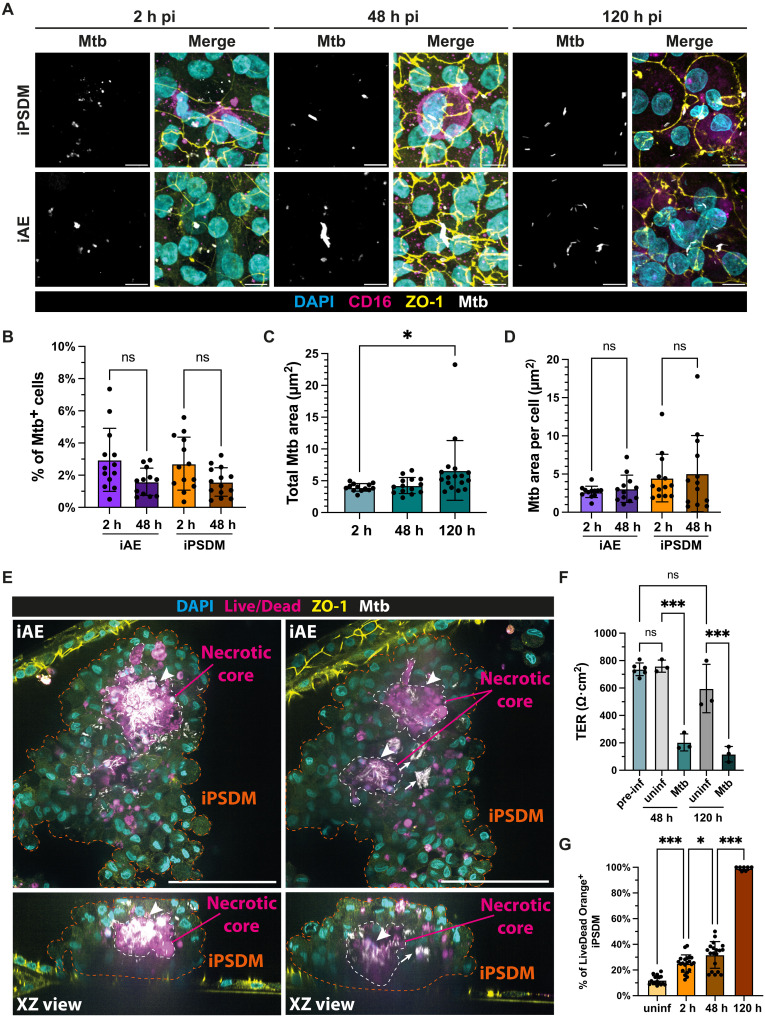
The iLoC mimic early stages of infection with Mtb. (**A**) Representative images of Mtb replication in iAE and iPSDM in iLoC at 2, 48, and 120 hours (h) pi under static condition, images showing nuclei (cyan), CD16 (magenta), ZO-1 (yellow), and Mtb (white). Scale bar, 10 μm. (**B**) Quantification of Mtb trophism in iAE and iPSDM in iLoC at 2 and 48 hours under static condition; means ± SD, *n* = 12 to 13 analyzed FOVs from *n* = 3 independent, one-way ANOVA. (**C**) Quantification of total Mtb area in iLoC at 2, 48, and 120 hours pi in iLoC under static condition; means ± SD, *n* = 12 to 21 analyzed FOVs from *n* = 3 independent experiment, one-way ANOVA. (**D**) Quantification of Mtb area in iAE and iPSDM at 2 and 48 hours pi in iLoC under static condition; means ± SD, *n* = 9 to 17 analyzed FOVs from *n* = 3 independent experiment, one-way ANOVA. (**E**) Representative confocal and XZ orthogonal images from two separate *z* planes showing the Mtb-induced macrophage aggregation and formation of a necrotic core (positive for dead staining). (**F**) TER measurement of iLoC throughout the course of Mtb infection under static conditions; *n* = 9 technical replicates from *n* = 3 independent experiment, one-way ANOVA. (**G**) Quantification of Live/Dead Orange-positive iPSDM in uninfected, 2, 48, and 120 hours pi under static condition; means ± SD, *n* = 7 to 21 analyzed FOVs from *n* = 3 independent experiment, one-way ANOVA. *P* value: ns *P* ≥ 0.05, *P < 0.05, ***P < 0.001.

To mimic alveolar tissue responses after Mtb infection, we aimed for a very low multiplicity of infection (MOI: 0.01; see Materials and Methods). Our choice of using a very low bacterial number compared to conventional in vitro studies was to achieve a closer mimicry of the physiological conditions of infection while maintaining experimental tractability (2000 bacteria per iLoC). Upon Mtb infection from the apical side of iLoC, we observed infection of both iPSDM and iAE by Mtb but not iVEC (Fig. 5A and fig. S18, A and B). Comparable levels of infection represented by the percentage of infected cells, total Mtb area, and Mtb area per cell were detected in macrophages and epithelial cells of iLoC at 2 hours postinfection (pi) (Fig. 5, B to D). Considering the ratio of iPSDM to iAT2 and iAT1 in the iLoC, our findings agree with previous reports that infection predominantly takes place in macrophages in conjunction with other alveolar cell types (*16*, *54*). In contrast to conventional 2D in vitro models, where Mtb replicates within 48 hours (*36*, *55*), most of the intracellular Mtb in both iPSDM and iAE did not substantially replicated within the first 48 hours of infection (Fig. 5, B to D).

Cell death is a hallmark of early TB and serves a key role in local inflammation and tissue damage. Strikingly, we noticed the rare occurrence of large macrophage clusters containing necrotic core-like structure and Mtb replication with rounded cellular morphology and clustering of iPSDM, suggesting that there are restricted focal areas where Mtb replicated (Fig. 5E). Moreover, there was a reduction but not a complete loss of barrier integrity as measured by TER at 48 hours pi, suggesting tissue damage but not destruction of the barrier (Fig. 5F). However, as the infection progressed to 120 hours pi, we observed a further decline in TER accompanied with the destruction of both epithelial and endothelial barriers, indicating a collapse of the alveolar barrier function (Fig. 5F and fig. S7A). Owing to the disruption of ZO-1 barriers of iLoC at 120 hours pi, the cell segmentation approach with ZO-1 used for earlier infection time points was no longer suitable for reliable cell segmentation. To extract the Mtb area in iLoC, we applied an alternative quantification approach to extract the total Mtb area in iLoC, where we observed comparable Mtb area at 48 hours and an increment of Mtb area at 120 hours after infection (Fig. 5C). Using a live/dead staining assay to evaluate the incidence of cell death in iLoC, we detected cell death prevalently in iPSDM within the first 48 hours of infection. In agreement with the observed loss of TER, we observed cell death in both iPSDM and iAE with disrupted epithelial barrier as the infection progressed to 120 hours pi (Fig. 5, F and G, and fig. S19, A and B). Together, the iLoC shows that during infection, both macrophages and epithelial cells are infected without substantial replication. However, we observed areas of cell death aggregation and Mtb replication that progressed to a severe disruption of both the epithelial and endothelial barrier.

### A GE-iLoC reveals cell type-specific autophagy responses in the alveolus

**Fig. 6.**
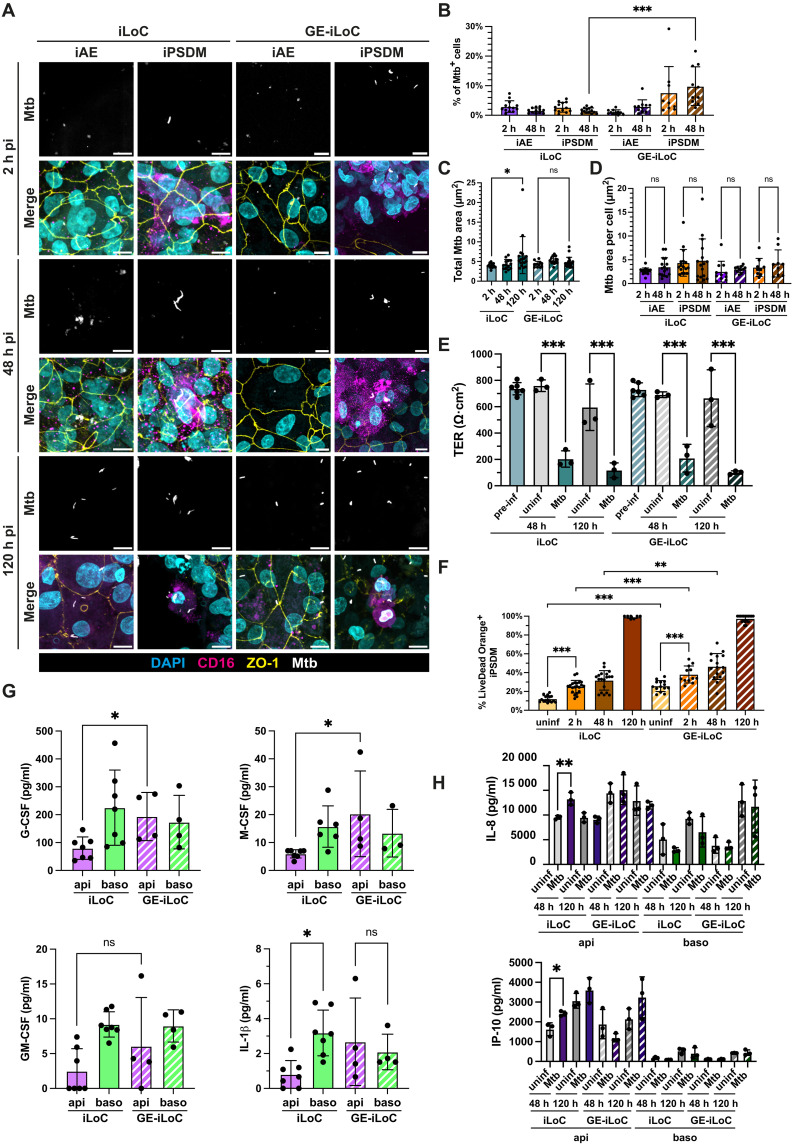
A GE-iLoC reveals cell type-specific autophagy responses in the alveolus. (**A**) Representative images of Mtb replication in iAE and iPSDM in iLoC or GE-iLoC at 2, 48, and 120 hours (h) pi; images showing nuclei (cyan), CD16 (magenta), ZO-1 (yellow), and Mtb (white). Scale bar, 10 μm. (**B**) Quantification of Mtb trophism in iAE and iPSDM in iLoC or GE-iLoC at 2 and 48 hours; means ± SD, *n* = 9 to 13 analyzed FOVs from *n* = 3 independent, one-way ANOVA. (**C**) Quantification of total Mtb area in iLoC or GE-iLoC at 2, 48, and 120 hours pi in iLoC or GE-iLoC; means ± SD, *n* = 12 to 21 analyzed FOVs from *n* = 2 to 3 independent experiment, one-way ANOVA. (**D**) Quantification of Mtb area in iAE and iPSDM at 2 and 48 hours pi; means ± SD, *n* = 9 to 17 analyzed FOVs from *n* = 3 independent experiment, one-way ANOVA. (**E**) TER measurement of iLoC and GE-iLoC throughout the course of Mtb infection under static conditions; *n* = 9 technical replicates from *n* = 3 independent experiment, one-way ANOVA. (**F**) Quantification of Live/Dead Orange-positive iPSDM WT and *ATG14KO* in uninfected, 2, 48, and 120 hours pi of Mtb infection; means ± SD, *n* = 7 to 21 analyzed FOVs from *n* = 2 to 3 independent experiment, one-way ANOVA. (**G**) Quantification of G-CSF, M-CSF, GM-CSF, and IL-1β secretion from the epithelial and endothelial side of uninfected iLoC and GE-iLoC; means ± SD, *n* = 4 to 7 independent experiment, one-way ANOVA. (**H**) Quantification of IL-8 and IP-10 secretion from the epithelial and endothelial side of iLoC and GE-iLoC in uninfected and infected conditions at 48 and 120 hours pi under static condition; means ± SD, *n* = 3 independent experiment, one-way ANOVA. *P* value: ns *P* ≥ 0.05, *P < 0.05, **P < 0.01, ***P < 0.001.

Autophagy is a key cellular degradative process, including pathogens via a lysosome-mediated degradation. In macrophages, autophagy is part of the innate immune response that restricts Mtb (*56*). In vitro, the autophagy protein ATG14 is required for the control of Mtb by human macrophages (*55*). To study the role of ATG14 in the iLoC, we generated a genetically engineered iLoC (GE-iLoC) consisting of *ATG14KO* iPSDM and wild-type (WT) iAT2, iAT1, and iVEC to define the role of ATG14 in macrophages in a physiologically relevant microenvironment. Upon Mtb infection from the apical side of iLoC, we observed infection of iPSDM and iAE by Mtb but not iVEC in the GE-iLoC, similar to the iLoC (Fig. 6A and fig. S18, A and B). Comparable levels of infection represented by the percentage of infected cells, total Mtb area, and Mtb area per cell were analyzed in macrophages and epithelial cells GE-iLoC at 2 hours pi (Fig. 6, B to D). We detected a larger fraction of infected macrophages in GE-iLoC as compared to iLoC at 48 hours after infection, suggesting enhanced bacterial uptake within the first 48 hours of infection (Fig. 6B). In contrast to conventional 2D in vitro models, where Mtb replicates within 48 hours (*36*, *55*), most of the intracellular Mtb in both iPSDM and iAE did not replicate within the first 48 hours of infection in the *ATG14KO* iPSDM in GE-iLoC (Fig. 6, A, C, and D). Moreover, macrophage clustering increased upon Mtb infection in the GE-iLoC (fig. S6, A and B). When the total Mtb area was measured, we observed a small but statistically significant increment of Mtb area at 120 hours pi in iLoC but not GE-iLoC (Fig. 6C). Notably, we observed an early disruption of the endothelial barrier in GE-iLoC when compared to the iLoC at 48 hours after infection, while disruption of both epithelial and endothelial barriers at 120 hours after infection (Fig. 6E and fig. S7A). Cell proliferation was similar in both the iLoC and the GE-iLoC when measured by Ki67 staining. These data suggest a macrophage-dependent role for ATG14 in endothelial damage but not cell proliferation (figs. S7 and S20, A and B). Cell death was induced by Mtb infection as early as 2 hours and *ATG14KO* macrophages showed higher basal as well as Mtb-induced cell death (Fig. 6F and fig. S19A). Both WT and *ATG14KO* macrophages harbored comparable load of intracellular Mtb, while *ATG14KO* macrophages showed higher cell death levels, suggesting a bacterial load independent trigger of macrophage cell death (Fig. 6, D and F). To further understand the influence of an ATG14-deficient macrophage to the alveolar microenvironment, we profiled the iLoC apical and basal cytokines. We observed higher levels of apical G-CSF and M-CSF as well as a loss of polarized secretion of IL-1β in the GE-iLoC, suggesting a remodeling of the alveolar microenvironment by *ATG14KO* macrophages (Fig. 6G). Upon Mtb infection, we detected elevated secretion of IL-8 and IP-10 at 48 hours pi in the iLoC but not in the GE-iLoC, indicating that ATG14 deficiency in macrophages alters alveolar inflammatory responses (Fig. 6H). Together, these data show that in the iLoC with autophagy-deficient macrophages, infection with Mtb triggered higher macrophage cell death without prominent bacterial replication, where the macrophage-specific autophagy deficiency also led to the remodeling of alveolar microenvironment in both uninfected and Mtb-infected conditions.

## DISCUSSION

This study is a report of a genetically engineered lung-on-chip that combines four isogenic human iPSC-derived cell types in an alveolar chip model (*57*). We built an autologous iLoC system consisting of iAT1, iAT2, iVECs, and alveolar macrophages with the capacity to incorporate CRISPR-Cas9 gene edited cellular components. Despite the successful recreation of an isogenic alveolar microenvironment, our system only represents a starting point for future improvements to the adoption of 3Rs principles (replacement, reduction, and refinement). Despite a high degree of lung cellular diversity, our alveolus-on-chip still requires further optimization to fully recapitulate the human alveolus. Our chip system enabled mechanical stretching of the key alveolar cell types yet lacked fluidic flow; future efforts shall evaluate the impact of fluidic flow on our cellular components. Here, we profiled the impact of endothelial cells on alveolar epithelium and macrophages. Future work will define the role of alveolar macrophages interactions that affect barrier integrity (Fig. 3B). The iLoC will allow incorporation of additional cell types such as fibroblasts and neutrophils to better recreate the diversity and heterogeneity of alveolar cells and improve the physiological relevance. In addition, each of the cell types in the iLoC was differentiated using one differentiation protocol from previously published protocols. Further efforts in protocol development will mimic the heterogeneity of iPSC-derived cells. For instance, we differentiated iPSDM from iPSDMono using GM-CSF. Alternatively, the ALI cocktail containing Infasurf, GM-CSF, transforming growth factor-β (TGF-β), and IL-10 (*47*) could potentially generate phenotypically different iPSDM due to the prolonged exposure to TGF-β and IL-10 that are also present in iLoC. Furthermore, the assembly of the iLoC is complex and time sensitive because of the current lack of long-term storage capabilities. In this context, cryopreservation approaches that offer good cell recovery and preservation of cellular phenotypes will broaden the system’s versatility and scalability.

We harnessed the self-assembly capacity of iLungPro to generate different lung epithelial cell types without an enrichment step after CPM^+^ iLungPro sorting. We generated a fraction of the intended alveolar epithelial cell types at an abundance comparable to the most recent reports in the field on cells before sorting (*28*) while achieving a good specification to the lung lineage and the cohabitation of iAT2 and iAT1 including transitional and proliferating cell states. Such phenomenon suggests that our epithelial cells have retained their proliferative capacity and that the iLoC could represent a therapeutic assessment model of lung damage and regeneration. Considering the lower expression of key alveolar cell type markers as compared to the others (*21*, *28*, *29*), future efforts are needed to address the long-term preservation of cell phenotypes in the iLoC. We observed in the iLoC differences in cytokine secretion essential for macrophage differentiation (M-CSF, GM-CSF, and G-CSF) (*42*, *45*) as well as inflammatory response (IL-6, IL-8, IL-1α, and IL-1Rα) (*43*–*45*). This secretion is modulated by endothelial cells, mechanical stretching, and ATG14-deficient macrophages. These differences could represent a differing ground state of cells, and further phenotypic and functional characterization at single-cell level will be needed. Using single-cell transcriptomics, we captured the impact of mechanical stretching and endothelial signaling on the alveolar cells. We found that mechanical stretch affected cell responses in a cell type-specific manner. Endothelial signaling appears to play diverse roles in the alveolar microenvironment, including induction of iAT2 and iAT1 differentiation, macrophage retention, global up-regulation of inflammatory genes, and antiviral pathways in epithelium and macrophages. Unexpectedly, the presence of endothelium in the iLoC leads to the enrichment of unique macrophage populations. These macrophages show stronger immune pathway up-regulation, further highlighting the significance of endothelium in tissue immunity and the need of multicellular system to mimic physiological conditions.

TB early innate immune responses are challenging to mimic in 2D cellular and animal models because of the limited physiological resemblance and accessibility (*58*). Applying the iLoC as an early model of TB, we observed Mtb infection of macrophages and alveolar epithelial cells as previously reported (*16*, *54*). In the iLoC, macrophages and epithelial cells are not permissive for Mtb replication as compared to 2D in vitro cultures. Strikingly, stochastic replication is observed together with macrophage cell death up to 2 days after infection. Such phenomenon can reflect the natural heterogeneous encounter of Mtb with macrophages in the alveolar space. The iLoC displayed inflammatory cytokine secretion and barrier function deterioration in response to Mtb infection, reflecting the immunoresponsiveness of iLoC for early disease modeling. As the infection progressed to 5 days, we observed a rapid decline in barrier function, epithelium disruption, and cell death. The observed phenomenon in the iLoC is different from previous observations that alveolar macrophages are less restrictive than blood-derived or bone marrow-derived macrophages (*47*, *59*). Such discrepancy could be attributed to differences in anatomical and genetic make-up between human and murine as well as the cell-cell interactions during Mtb infection. Our data suggest the iLoC represents an alveolar system displaying strong inflammatory responses and tissue damage. Further studies shall address the underlying molecular drivers and the impact on TB pathogenesis.

Using a genetically engineered *ATG14KO* hybrid GE-iLoC, we found that ATG14-deficient macrophages are more susceptible to cell death under homeostatic and Mtb-infected conditions. We observed higher bacterial uptake by macrophages from GE-iLoC, different from macrophage 2D monoculture systems, and further investigation shall shed light on the pathophysiological implications of such phenomenon. Considering the minimal Mtb replication and increase of infected macrophages in the first 48 hours after infection in GE-iLoC, future efforts using live microscopy to capture the spatiotemporal dynamics of the infection will define the source of bacteria that led to elevated macrophage infection. After infection of the iLoC, we observed an elevated secretion of cytokine predominantly at the apical chamber, which could be a consequence of infection that took place at the apical chamber at the earlier phase of infection. An elevation of cytokine secretion at the basolateral chamber could subsequently take place to recruit immune cells from the basal circulation, and this will be considered in the next generation of the iLoC. There was an elevation of IL-8 and IP-10 in the iLoC but not GE-iLoC after infection, suggesting that when added, other immune cells could be recruited. In this context, IL-8 and IP-10 recruit and activate neutrophils, monocytes, T cells, eosinophils, and natural killer cells (*45*). The observed differences in the GE-iLoC bring forward the potential unstudied link between autophagy deficiency at cellular level and severe tissue pathologies. Extracellular mycobacterial cording has been observed in other lung-on-chip models (*15*), and we did not observe this predominately in our system, which could be attributed to multiple factors that differ between the two reported systems, including but not limited to cell sources, cellular physiology, microenvironmental cues, and Mtb strains.

Respiratory infections and complications are among the top killers of human and major public health concerns. The development of physiologically relevant model systems as the one described here is important to understanding the fundamental biology of respiratory diseases. Here, we report the building of the iLoC and its capacity to study early events in TB. This system can be applied to modeling and drug discovery of other pulmonary diseases.

## MATERIALS AND METHODS

### Human iPSC culture and maintenance

KOLF2HPSI0114i-kolf_2 (KOLF2) human iPSC was sourced from Public Health England Culture Collections (catalog number 77650100). *ATG14KO* KOLF2 iPSC were generated as described before (*55*). To resume iPSC culture from cryopreserved stocks, six-well tissue culture-treated plates are coated with Vitronectin XF (STEMCELL Technologies), diluting 40 μl in 1 ml of phosphate-buffered saline (PBS, Thermo Fisher Scientific), incubated at room temperature for 1 hour), and then the Vitronectin solution is replaced with 1 ml of Essential 8 medium (hereafter E8, Thermo Fisher Scientific) + 10 μM Y-27623 (Tocris Bioscience) per well. Cryopreserved vials of iPSC were thawed in 37°C water bath, and contents are transferred to 10 ml of E8 in a 15-ml centrifuge tube. iPSC is collected by centrifugation at 300*g* for 3 min and plated in Vitronectin-coated plates in E8 + 10 μM Y-27632. From the following day onward, the medium is changed to E8 without Y-27632 daily until confluent. To maintain iPSC culture, iPSC is washed with PBS and dissociated with Versene (Thermo Fisher Scientific, incubated at room temperature for 5 min). The Versene is then removed, and iPSC is resuspended to a suspension of small clumps in E8 or mTeSR Plus medium (STEMCELL Technologies). The iPSC is then split in a ratio of 1:6 into plates coated with Matrigel (Corning, according to the manufacturer’s guide) in mTeSR Plus. iPSC cultures are passaged every 3 to 5 days using Gentle cell dissociation buffer (STEMCELL Technologies) or Versene.

### Alveolar epithelial cells differentiation from human iPSC

iPSC were seeded at 2 × 10^6^ cells per well in Matrigel-coated six-well plate in mTeSR Plus + 10 μM Y-27632. iPSC were cultured for 3 days as a monolayer in a chemically defined medium (STEMdiff definitive endoderm kit, STEMCELL Technologies) to induce the commitment of the cell population to the definitive endoderm state. Successful differentiation is indicated by a high incidence (>80%) of CXCR4^+^CKIT^+^ analyzed by flow cytometry. In the following differentiation steps, cells are cultured in base medium cSFDM [3:1 v/v mix of Iscove’s modified Dulbecco’s medium (IMDM) and Dulbecco’s modified Eagle’s medium (DMEM)/Ham’s medium F12 mix, 1% GlutaMAX, 0.5% N-2 supplement, 1% B-27 supplement, 0.05% bovine serum albumin (BSA), ascorbic acid (50 μg/ml), 0.45 mM 1-Thioglycerol, and primocin (200 ng/ml)] (*23*). Definitive endoderm cells are replated and cultured for 3 days in TGF-β/BMP4 inhibiting AFE medium (2 μM dorsomorphin, Selleck Chemicals; 10 μM SB431542, Selleck Chemicals) to commit to anterior foregut endoderm. Next, cells are cultured for 4 days in vAFE medium [rhBMP4 (20 ng/ml), Peprotech; 3.5 μM CHIR99021, Selleck Chemicals; and 1 μM retinoic acid, Merck] to achieve ventralization. Subsequently, the cells are cultured for 7 days in progenitor-specifying LungPro medium (recombinant human keratinocyte growth factor (KGF) (10 ng/ml), R&D Systems Inc.; 20 μM γ-secretase inhibitor {N-[N-(3,5-difluorophenacetyl)-L-alanyl]-S-phenylglycine t-butyl ester; DAPT}, Merck; fibroblast growth factor 10 (10 ng/ml), PeproTech; and 3 μM CHIR99021) to derive NKX2-1^+^ lung progenitor cells.

Lung progenitors are dissociated using TrypLE (Thermo Fisher Scientific, 10 to 15 min at 37°C), and NKX2-1^+^ lung progenitor cells are enriched by fluorescence-activated cell sorting (FACS) using surrogate cell surface marker CPM. Enriched CPM^+^ lung progenitor cells are plated in Matrigel-coated AX12 plates at a density of 9.7 × 10^5^ cells/cm^2^ (a seeding density that enabled rapid coverage of AX12 membrane and iAE barrier formation) in alveolar medium (10 ng/ml recombinant human KGF; 50 μM dexamethasone, Merck; 0.1 mM 8-bromoadenosine 3′:5′-cyclicmonophosphate, Merck; 0.1 mM 3-isobutyl-1-methylxanthine, Merck; and 10 μM Y-27632) for 7+ days to yield alveolar epithelial cells. The apical medium is removed to form ALI to trigger iAT2 to iAT1 transdifferentiation (*27*).

### Alveolar macrophage differentiation from human iPSC

For embryonic body (EB) formation using AggreWell 800 (STEMCELL Technologies), AggreWell is treated with Anti-Adherence rinsing solution (STEMCELL Technologies), centrifuged for 5 min at 300*g* and then washed with PBS and preincubated with E8 + 10 μM Y-27632. iPSC is harvested and plated onto AggreWell at 4 × 10^6^ cells per well with centrifugation at 100*g* for 6 min. For the following 3 days, the medium was changed daily to fresh EB medium [E8, BMP4 (50 ng/ml), vascular endothelial growth factor (50 ng/ml, VEGF-A, PeproTech, #100-20), and stem cell factor (20 ng/ml, SCF, PeproTech, #300-07]. To set up EB factories, EBs are harvested from AggreWell by pipette up and down and collected through a 40-μm cell strainer and seeded into tissue culture-treated flask in factory medium [X-VIVO 15, Lonza; 2 mM GlutaMAX; 50 μM β-Mercaptoethanol, Gibco; M-CSF (100 ng/ml, PeproTech); and IL-3 (25 ng/ml, PeproTech)]. For factory maintenance, the factory is fed weekly with factory medium. The factories begin to produce monocytes 4 to 5 weeks postestablishment. For macrophage differentiation, monocytes are collected from the factory supernatant by centrifugation at 300*g* for 5 min and plated into 10-cm dishes at 5 × 10^6^ cells per dish in differentiation media (X-VIVO 15; 2 mM GlutaMAX; and GM-CSF (50 ng/ml, PeproTech)]. The seeded cells are boosted with fresh differentiation medium 3 or 4 days postseeding. Depending on the demand of macrophages, this process can be scaled up or down (*36*).

### Endothelial cell differentiation from human iPSC

iPSCs were seeded at 700,000 cells per plate in Matrigel-coated 10-cm dish in mTeSR Plus + 10 μM Y-27632, and the medium was replaced with mTeSR Plus on the following day. iPSC were cultured for 3 days as a monolayer in a chemically defined medium N2B27 [50% DMEM/F12 medium, Gibco; 50% Neurobasal medium, Life Technologies; 2X B-27 supplement; 1X N-2 supplement; 0.1% β-Mercaptoethanol; 8 μM CHIR99021; and rhBMP4 (25 ng/ml)] to induce the commitment toward lateral mesodermal state. The cells are then cultured for 2 days in VEC medium [StemPro-34 SFM, Life Technologies; 1X GlutaMAX; VEGF-A (200 ng/ml); and 2 μm forskolin, Abcam] to achieve differentiation into endothelial cells. The endothelial cells are harvested and CD144^+^ endothelial cells are enriched using magnetic cell sorting.

### Multicellular iLoC assembly

A complete diagram of the assembly of the iLoC is shown in Fig. 4A.

#### Step 1: iVEC sorting and seeding

Differentiated cells were detached, magnetically enriched for CD144^+^ iVECs, and counted according to “Endothelial cell differentiation from iPSC” and “iVEC enrichment by magnetic cell sorting” protocols. iVECs (8 × 10^4^) are seeded in 15 μl of iVEC medium per membrane on basolateral side of AX12. iVECs are allowed to attach overnight (under unclosed condition) at 37°C, 5% CO_2_. On the next day, the AX12 plate is initialized with iVEC medium [StemPro-34 SFM; VEGF-A (50 ng/ml); and 10 μM Y-27632].

#### Step 2: iLungPro sorting and seeding

Differentiated cells are detached, enriched for CPM^+^ iLungPro according to “Alveolar epithelial cell differentiation from iPSC” and “iLungPro enrichment by FACS” protocol. iLungPro (8 × 10^4^) are seeded in 70 μl of alveolar medium per membrane on the apical side of AX12. iLungPro are allowed to attach overnight at 37°C, 5% CO_2_.

#### Step 3: Seeding of iPSDM

To detach differentiated iPSDM, cells are incubated with Versene, lifted using a lifter, collected by centrifugation, and counted. To achieve a concentrated cell suspension for iPSDM seeding (2 × 10^6^ iPSDM/ml), desired amount of iPSDM is collected by centrifugation and resuspended in small volume of iLoC medium to 2 × 10^6^ iPSDM/ml. Ten microliters of iPSDM cell suspension is added to each AX12 membrane to achieve an iPSDM:iAT2 and iAT1 ratio of 1:5-1:10.

We have generated iLoC using this protocol using three iPSC lines to date, including WTSIi018-B (RRID:CVCL_AE29), CRICKi018-A (RRID:CVCL_D0DD), and CRICKi019 (RRID:CVCL_D0DE). In generating the figures of this work, we used the iPSC line WTSIi018-B, derived from a healthy donor.

### Maintenance of the iLoC

From day 2 onward, apical and basal media are changed to fresh alveolar medium and iVEC medium, respectively, every 2 days until the formation of ALI. Typically, a TER reading of ≥200 Ω·cm^2^ is detectable on day 4 postseeding of iLungPro. To initiate 3D breathing after reaching a TER reading ≥200 Ω·cm^2^, the AX12 plate is placed into the ^AX^Dock connected to the ^AX^Breather (all components provided by AlveoliX) and closed tight, and 3D breathing of the desired chip is initiated. The iLoC is subjected to a 3D surface strain of 16.6% (translated to a linear strain of 8%) at 0.2 Hz (*8*). To form ALI, the apical medium is removed, and the basal medium is switched to iLoC medium (50% alveolar medium; 50% iVEC medium) in a reduced volume to balance the hydrostatic pressure between the wells according to the manufacturer’s instructions. After ALI formation, the basal medium is changed to fresh iLoC medium every 2 days. A detailed description on the design and handling of ^AX^Lung-on-Chip System has been reported recently (*14*).

### Immunostaining and microscopy

For cells seeded on glass coverslips, the cells were washed with PBS and fixed with 4% paraformaldehyde (PFA, diluted from 16% stock, Electron Microscopy Sciences) in PBS for 15 min at room temperature. For cells seeded on AX12, half of the apical medium was removed and equal volume of 8% PFA in PBS is added; 4% PFA in PBS is flowed into the basolateral chamber to fix the cells for 15 min at room temperature. For all samples infected with Mtb, the samples were fixed in 4% PFA in PBS (final concentration) at 4°C overnight to ensure complete killing of Mtb. The fixed cells were then washed three times with PBS and stored in 1% BSA PBS at 4°C.

Cells were permeabilized with 0.01% saponin (Merck) in 1% BSA (Cell Signaling Technology) PBS for 1 hour at room temperature and then blocked in 3% BSA PBS for 1 hour. The cells were washed three times with PBS and then stained with primary antibodies in 1% BSA PBS for 1 hour at room temperature. The cells were washed three times with PBS and then stained with 4′,6-diamidino-2-phenylindole (DAPI, Invitrogen) and secondary antibodies in 1% BSA PBS for 1 hour at room temperature. The cells are then washed three times with PBS and mounted using Dako Omnis Fluorescence Mounting Medium (Agilent), dried overnight at room temperature, and stored at 4°C. The stained samples were imaged using Olympus CSU-W1 SoRa Spinning Disk Microscope (Olympus) using 20× 0.7 numerical aperture (NA) air, 40× 1.25 NA, and 60× 1.3 NA silicone immersion objectives or STELLARIS 8 Confocal Microscope Platform (Leica Microsystems) using 40× 1.3 NA oil immersion objective.

### Flow cytometry

For characterization of cell surface markers, cells (2 × 10^5^ cells per marker) were washed once with PBS and detached using TrypLE. The cells were collected by centrifugation, washed with PBS, and stained with fluorochrome-conjugated antibodies in 1% BSA PBS on ice for 1 hour. The cells were washed with PBS, resuspended in 1% BSA PBS, and stored on ice before analysis. For intracellular staining, cells were fixed with 4% PFA in PBS for 15 min, washed with PBS, and permeabilized in Intracellular Staining Permeabilization Wash Buffer (BioLegend) for 1 hour. The cells were collected by centrifugation, washed with PBS, and stained with primary antibody on ice for 1 hour. The cells were washed with PBS and stained with fluorochrome-conjugated secondary antibodies in 1% BSA PBS on ice for 1 hour. The cells were washed with PBS, resuspended in 1% BSA PBS, and stored on ice before analysis. The cells were analyzed using a BD LSRFortessa Cell Analyzer (BD Biosciences) and analyzed using FlowJo software (FlowJo, LLC, v10.8.1). At least 10,000 events per condition were recorded.

### iLungPro enrichment by FACS

iLungPro were washed with PBS and detached using TrypLE at 37°C for 10 min. DMEM (Thermo Fisher Scientific) + 10% FBS (Thermo Fisher Scientific) was added to the dissociated cells inactivate to TrypLE. The cells were collected by centrifugation at 300*g* for 5 min and resuspended to achieve 10^7^ cells/ml and stained with anti-CPM antibody (FUJIFILM Wako Shibayagi, 1:200) in 10% FBS PBS on ice for 1 hour. The cells were then washed with PBS and stained with Cy3-conjugated secondary antibodies (Thermo Fisher Scientific) in 10% FBS PBS on ice for 1 hour. The cells were washed with PBS, resuspended in sorting buffer (10% FBS PBS; 10 μM Calcein blue, Life Technologies; and 10 μM Y-27632) and stored on ice before cell sorting. The cells were sorted using a BD FACSAria Fusion Flow Cytometer (BD Biosciences) to enrich CPM^+^ iLungPro cells into collection buffer (10% FBS PBS and 10 μM Y-27632) kept at 4°C. The sorting quality is checked before downstream processes.

### iVEC enrichment by magnetic cell sorting

iVEC were washed with PBS and detached using Accutase (STEMCELL Technologies) at 37°C for 5 min. DMEM/F12 (Thermo Fisher Scientific) was added to the dissociated cells to dilute out Accutase; the cells were collected by centrifugation at 300*g* for 5 min and resuspended to achieve 1.2 × 10^7^ cells/ml and stained with anti-CD144 microbeads in autoMACS Rinsing Solution (Miltenyi Biosciences) on ice for 20 min. The cells were washed with autoMACS Rinsing Solution and collected by centrifugation at 300*g* for 5 min and resuspended in 500 μl of autoMACS Rinsing Solution. The resuspended cells were passed through LS Column (Miltenyi Biosciences), and the retained cells were eluted in autoMACS Rinsing Solution + 10 μM Y-27632 for downstream culture.

### Reverse transcription quantitative PCR

After the cells were washed once with PBS and lysed in 350 μl RLT buffer (QIAGEN), the total RNA was extracted using RNeasy Mini Kit (QIAGEN). Total RNA was pooled from duplicate wells per condition and stored at −80°C until further use. The total RNA was measured using a NanoDrop One/OneC Microvolume UV-Vis Spectrophotometer (Thermo Fisher Scientific) to assess quality and yield. Conversion to cDNA was achieved using a QuantiTect Reverse Transcription Kit (QIAGEN). RT-qPCR analysis was performed using a QuantStudio 12 K Flex Real-Time PCR System (Thermo Fisher Scientific) and TaqMan probe-based detection (Thermo Fisher Scientific) in triplicate. The expression of target genes was normalized to house-keeping gene β-actin, and fold expression was calculated using ^∆∆^Ct method in comparison to enriched iLungPro cells or a primary adult lung sample.

### LDL uptake assay

iVECs were incubated with Alexa Fluor 594 AcLDL (2.5 μg/ml, Thermo Fisher Scientific) in StemPro34 medium for 4 hours at 37°C. Thereafter, the cells were washed three times with PBS, detached with TrypLE, and fixed with 4% PFA in PBS for 10 min at room temperature. The cells were analyzed using a BD LSRFortessa Cell Analyzer and analyzed using FlowJo software. At least 10,000 events per condition were recorded.

### Angiogenesis assay

Angiogenic potential was tested as described before (*60*). Briefly, tissue culture plate was coated with undiluted Matrigel at 150 μl/cm^2^, and iVECs were seeded at 3 × 10^4^ cells/cm^2^. The formation of tubular structure is inspected 6 hours post-iVEC seeding.

### TER measurements

TER measurements were used to assess the barrier integrity of epithelial and endothelial cells seeded on AX12. The TER measurements were measured using an Epithelial Volt/Ohm Meter (EVOM2 and EVOM3; World Precision Instruments) using a 96-well plate electrode (STX100 Electrode Corning 96; World Precision Instruments) with the connector (EVOM3 legacy probe kit, World Precision Instruments) for EVOM3. Twenty-four hours postepithelial seeding, TER measurements were taken every day until the initiation of the ALI where measurements were then taken every other day. To take the measurements, the electrode was sterilized with 70% v/v ethanol (Thermo Fisher Scientific) and connected to the EVOM2 or EVOM3. After securely placing the AX12 device into the ^AX^Dock, the “TEER Measurement” function was selected on the ^AX^Exchanger. The electrode was positioned between the outlet and central wells, and the resistance value was recorded. To measure the background TER, an empty AX12 plate without cells was used. The background value was subtracted from the measured value of a sample and then multiplied by the surface area of the well (0.071 cm^2^) to obtain the final TER reading in Ω·cm^2^.

### Scanning electron microscopy

Samples were fixed with a mixture of 4% formaldehyde, 1.25% glutaraldehyde (Merck), 0.04 M sucrose in 200 mM Hepes (Merck) (pH 7.4) overnight at 4°C. The samples were processed using a Biowave Pro (Pelco, USA) as follows: The samples were washed twice in 200 mM Hepes (pH 7.4) at 250 W for 40 s, postfixed/contrasted using a solution of 2% osmium tetroxide (Taab) and 1.5% potassium ferricyanide (Taab). The samples were washed with ddH_2_O and incubated with 1% thiocarbohydrazide (Merck) in distilled water (v/v) for 14 min at 100 W power (with/without vacuum 20 ″Hg at 2-min intervals). The samples were then incubated with 2% osmium tetroxide distilled water (w/v) for 14 min at 100 W power (with/without vacuum 20 ″Hg at 2 min intervals) and washed. The samples were incubated in 1% aqueous uranyl acetate (Agar scientific) in distilled water (w/v) for 14 min at 100 W power (with/without vacuum 20 ″Hg at 2-min intervals) and then washed. The samples were dehydrated using a stepwise ethanol series of 50, 75, 90, and 100% v/v at 250 W for 40 s per step. Critical point drying (CPD) was performed using an EM CPD300 (Leica Microsystems) and ethanol as the solvent. Samples were coated with 2-nm gold before imaging on a Quanta SEM (FEI, United States).

### Transmission electron microscopy

Samples were processed using a Biowave Pro (Pelco, USA) with use of microwave energy and vacuum. In the first instance, the samples were fixed by adding a double-strength mixture of 8% formaldehyde, 2.5% glutaraldehyde, and 0.04 M sucrose in 200 mM Hepes (pH 7.4) v/v to the apical side of the membrane. This was followed by a media exchange to replace the basal medium with mixture of 4% formaldehyde, 1.25% glutaraldehyde, and 0.04 M sucrose in 200 mM Hepes (pH 7.4). Samples were incubated at room temperature for 5 min before being transferred to fresh single strength fixative and allowed a further 30 min at room temperature before transfer and storage at 4°. The samples were washed twice in 100 mM Hepes at 250 W for 40 s and postfixed using a solution of 2% osmium tetroxide and 1.5% potassium ferricyanide. The samples were washed with ddH_2_O and treated with 1% thiocarbohydrazide in distilled water (v/v) for 14 min at 100 W power (with/without vacuum 20 ″Hg at 2-min intervals). The samples were incubated with 2% osmium tetroxide distilled water (w/v) for 14 min at 100-W power (with/without vacuum 20 ″Hg at 2-min intervals) and then washed. Samples were incubated in 1% aqueous uranyl acetate in distilled water (w/v) for 14 min at 100 W power (with/without vacuum 20 ″Hg at 2-min intervals) and then incubated with Reynolds’ lead aspartate for 14 min at 100-W power (with/without vacuum 20 ″Hg at 2-min intervals). Samples were infiltrated with a dilution series of 25, 50, 75, and 100% Durcupan ACM (Merck) (v/v) resin to ethanol. Each step was 3 min at 250-W power (with/without vacuum 20 Hg at 30-s intervals). Sample were cured for a minimum of 48 hours at 60°C. Sample block was orientated for trimming the cells transversely against the membrane. Using a razor blade, excess resin and plastic was removed, and then fine-trimming is done using a 35° ultrasonic, oscillating diamond knife (DiATOME, Switzerland) set at a cutting speed of 0.6 mm/s. The frequency is set by automatic mode, and a voltage of 6.0 V is applied. The knife is installed in a ultramicrotome EM UC7 (Leica Microsystems). 55- to 70-μm sections were transferred to TEM slot grids with a plioform support film and allowed to dry before imaging by TEM imaging using a JEOL1400FLASH/TEM. Micrographs were visualized and linear adjustments made to contrast and brightness using FIJI (version:2.9.0/1.54f).

### Image-based single-cell analysis

For the quantification of SP-C, PDPN, and ABCA3, the first step in this analysis was to automatically segment the full cytoplasmic extent of each cell. This was achieved by using a ZO-1 membrane marker as an input to the generalist segmentation algorithm Cellpose (*61*). A comprehensive, whole-cell measurement of fluorescent marker intensity was needed as the expression of SP-C/PDPN/ABCA3 was variable across the cell height in the *z* axis. To achieve this, the btrack (*62*) tracking algorithm was co-opted to link each single-cell instance in each *z* slice over the full image volume. During the initial localization step of the btrack method, the underlying single-cell mean intensities of the cell-type fluorescent markers were recorded for each segment. For manual verification of this analysis, a bespoke napari (*63*) key binding script was created that allowed for the unbiased selection of a subsample of user-chosen positive and negative single-cell examples of each dataset. This resulted in a distribution of pixel values for both positively and negatively expressing cells, resulting in a reliable quantification of single-cell identity on a population level that helped inform the veracity of this approach.

For the quantification of AGER, the image stacks were loaded into FIJI (version: 2.9.0/1.54f) using Bio-Formats Importer, and Z-projections were generated using maximum projection. An individual nuclei region of interest (ROI) was segmented by thresholding the DAPI channel and quantified as the total cell number. The AGER channel was thresholded to remove background signal, and cells with positive AGER signal on cell membrane contacting all neighboring cells were manually quantified.

For the quantification of nuclei per iPSDM cluster in iLoC, the image stacks were loaded into FIJI (version: 2.9.0/1.54f) using Bio-Formats Importer. iPSDM clusters were identified by cell morphology and the absence of ZO-1 marker; the number of nuclei per iPSDM cluster was manually quantified.

### Image-based single-nuclei analysis

The image stacks were loaded into FIJI (version: 2.9.0/1.54f) using Bio-Formats Importer, and Z-projections were generated using maximum projection. An individual nuclei ROI was segmented by thresholding the DAPI channel and converting the binary image mask to selection. NKX2-1 channel and NKX2-1 fluorescence intensity were measured using Analyze Particles, redirecting the analysis of the ROIs to the other image channels.

### Library preparation for scRNA-seq

To dissociate the apical cells, 70 μl of TrypLE was added to the apical chamber of AX12 and 200 μl of TrypLE is flowed into the basal chamber using “medium change” function of ^AX^Exchanger; AX12 was then incubated at 37°C for 10 min. The apical side was washed once with PBS and then dissociated to single cells by pipette up and down and transferred to 1% BSA PBS. AX12 is then opened and flipped upside down, and basal monolayer was dissociated to single cells by pipette up and down and transferred to 1% BSA PBS; the basal side was washed once with PBS and collected. The harvested single cells were collected by centrifugation at 300*g* for 5 mins at 4°C, washed once in PBS, and resuspended in 0.04% BSA PBS, passed through cell strainer (Bel-Art), and counted using acridine orange and propidium iodide and the Luna-FX7 Automatic Cell Counter (Logos Biosystems). Approximately 20,000 cells were loaded on Chromium Chip and partitioned in nanoliter-scale droplets using the Chromium Controller and Chromium Next GEM Single Cell Reagents [10x Genomics, CG000315 Chromium Single Cell 3’ Reagent Kits User Guide (v3.1 - Dual Index)]. Within each droplet, the cells were lysed, and the RNA was reverse transcribed. The resulting cDNA within a droplet shared the same cell barcode. Illumina compatible libraries were generated from the cDNA using Chromium Next GEM Single Cell library reagents in accordance with the manufacturer’s instructions [10x Genomics, CG000315 Chromium Single Cell 3’ Reagent Kits User Guide (v3.1 - Dual Index)]. Final libraries are QC’d using the Agilent TapeStation (Agilent Technologies) and sequenced using the Illumina NovaSeq 6000 Sequencing System (Illumina) using Sequencing read configuration: 28-10-10-90.

### Bioinformatic analysis of scRNA-seq

Gene expression was quantified using Cell Ranger count (v.6.0.1) against the prebuilt reference GRCh38-2020-A (10x Genomics). All subsequent analyses were carried out using Seurat (v4) package (*64*, *65*), with default parameters unless specified, in R-4.2.0 [R Core Team (2022) https://R-project.org/]. Each individual dataset (iAM breathing, iAM static, iLoC breathing, and iLoC static) was normalized with the “LogNormalize” method. The top 2000 highly variable genes were identified using the “FindVariableFeatures” function, and the data were scaled (“ScaleData” function). We run principal components analysis selecting the first 20 principal components (PCs), constructed a shared nearest neighbor graph using the “FindNeighbors” function and clustered the cells using Louvain clustering (“FindClusters” function) at a resolution of 1.2. Clusters with high average number of mitochondrial content (>5 percent.mt), low average number of genes (<1500 nFeature_RNA), and low number of molecules (<5000 nCount_RNA) were removed, and individual samples were reprocessed as before, recalculating the highly variable features and selecting the first 20 PCs to construct Uniform Manifold Approximation and Projection (UMAP) plots. All four individual datasets were then integrated (“IntegrateData” function) using the Seurat standard CCA integration workflow, after identifying 2000 anchor features. The resulting integrated data were visualized on the UMAP space using the first 50 PCs. Integrated clusters at resolution 1 were annotated on the basis of a combination of the expression of known marker genes for each cell type, and top cluster-specific gene markers identified using the “FindAllMarkers” function (with the settings: only.pos = TRUE, logfc.threshold = 0.25, min.pct = 0.2). The integrated dataset was also compared to a reference Human Lung Cell Atlas of the healthy respiratory system spanning more than 584,000 cells of 61 cell identities (*46*). To assess similarities between them, we used (i) the cell label transfer method and projection of query cells onto reference UMAP structure (“FindTransferAnchors” and “MapQuery” functions) from Seurat, and (ii) the cluster label assignment from “clustifyr” (v1.10.0) that adopts a Spearman correlation-based method to find reference transcriptomes with the highest similarity to query cluster expression profiles (*66*). The iPSDM population identified in the integrated data was also subsetted for further investigation. iPSDM cells were extracted from the individual samples, reprocessed, and integrated again as described above and subclustered at resolution 0.6. Differential expression analysis was performed using the “FindMarkers” function (DESeq2 test without logfc or minimum percentage expressed thresholds). The Seurat DESeq2DETest function was modified to output the Wald statistic. Gene Set Enrichment Analysis (*67*) was performed using “ClusterProfiler” v.4.6.2 (*68*). Gene lists from the differential expression analysis for each comparison were ranked by the Wald statistic. C2-CP:REACTOME from the MSigDB collection (MSigDB v2023.1) was assessed with the parameters minGSSize = 5 and maxGSSize = 5000, and only pathways with an adjusted *P* value lower than 0.05 were considered statistically significant (*69*). Dot plots illustrating selected pathways were made with ggplot2.

### Cytokine measurements

Apical and basal media of AX12 were harvest and centrifuged through 0.22-μm filter column (Thermo Fisher Scientific) twice to remove cells and Mtb. The media were stored at −80°C before cytokine analysis. Cytokine screening was performed using Bio-Plex Pro Human Cytokine Screening Panel, 48-Plex (Bio-Rad) on a Bio-Plex 200 System (Bio-Rad) following the manufacturer’s protocol.

### Mtb infection

Mtb H37Rv cryovials were thawed and cultured to late log phase in Middlebrook 7H9 broth medium (BD Biosciences) with hygromycin B (50 μg/ml, Thermo Fisher Scientific). The Mtb culture is collected by centrifugation at 3000*g* for 5 min, washed twice with PBS, and collected again by centrifugation. The bacterial pellet is dissociated by vortexing with glass beads for 1 min. The dissociated bacteria were resuspended in Alveolar medium and centrifuged at 1200*g* for 5 min. The supernatant containing single bacterium are collected and measured using a spectrophotometry at optical density at 600 nm. The Mtb is diluted in alveolar medium and infected the iLoC from apical side aiming to achieve MOI = 0.01 without washout until experimental end point. After infection, apical and basal media were harvested and the iLoC were fixed as designated time points with 4% PFA in PBS at 4°C overnight.

### Quantification of Mtb in iLoC

For the quantification of Mtb in iAE and iPSDM at infection time points of 2 and 48 hours pi (i.e., intact ZO-1 barrier), the iAE were automatically segmented using a ZO-1 membrane marker as input to Cellpose (*61*) and the iPSDM were manually segmented using napari (*63*). The Mtb channel was set with an intensity threshold to visualize only the bacteria, and the Mtb area per segmented cell were then measured. For the quantification of total Mtb in iLoC at infection time point of 2, 48, and 120 hours pi, the image stacks were loaded into FIJI (version: 2.9.0/1.54f) using Bio-Formats Importer, and Z-projections were generated using maximum projection. Individual Mtb ROI was segmented by thresholding the Mtb channel, converted to mask, and the ROI areas were measured using Analyze Particles.

### Live/Dead Orange staining of iLoC

Thirty minutes before the designated time point of iLoC sampling, LIVE/DEAD Fixable Orange (602) Viability Kit (Thermo Fisher Scientific) was added to apical and basal media. At designated time points, iLoC were harvested as described above. Harvested samples were permeabilized with 0.01% saponin in 1% BSA PBS for 1 hour at room temperature and then blocked in 3% BSA PBS for 1 hour. The cells were washed three times with PBS and then stained with DAPI (1:10000) and Alexa Flour 488 anti-ZO-1 antibody (Thermo Fisher Scientific, #339188, 1:100) in 1% BSA PBS for 1 hour at room temperature. Cells are then washed three times with PBS and mounted using Dako Omnis Fluorescence Mounting Medium, dried overnight at room temperature, and stored at 4°C. The stained samples were imaged using Olympus CSU-W1 SoRa Spinning Disk Microscope using 20× 0.7 NA air objectives, and LIVE/DEAD-positive cells were manually quantified.

### Quantification and statistical analysis

All data presented in this work were obtained from at least *n* = 3 independent experiments. Statistical analyses applied are described in each figure legend. For the comparison of two groups, unpaired Student’s *t* test was used (fig. S4, C and D). For the comparison of multiple groups with one variable, one-way analysis of variance (ANOVA) with Tukey’s post hoc test was used (Figs. 1, D and I, 3G, 5, B to D, and 5, F to H). *P* < 0.05 was considered statistically significant. Statistical significance was determined using Prism v.10.0.1 software (GraphPad).
