# Supplementary material for: Autologous human iPSC–derived alveolus-on-chip reveals early pathological events of Mycobacterium tuberculosis infection
Source: Sci Adv. 2026 Jan 1;12(1):eaea9874. doi: 10.1126/sciadv.aea9874 (PMC12757024; doi:10.1126/sciadv.aea9874)
Supplement: Supplementary file 1 — Figs. S1 to S20 Table S1 Legend for data S1 [file sciadv.aea9874_sm.pdf]

Science Advances
AAAS

Supplementary Materials for

Autologous human iPSC–derived alveolus-on-chip reveals early pathological events of *Mycobacterium tuberculosis* infection

Chak Hon Luk *et al.*

Corresponding author: Chak Hon Luk, jakson.luk@crick.ac.uk; Maximiliano G. Gutierrez, max.g@crick.ac.uk

Sci. Adv. **12**, eaea9874 (2026)
DOI: 10.1126/sciadv.aea9874

The PDF file includes:

Figs. S1 to S20
Table S1
Legend for data S1

Other Supplementary Material for this manuscript includes the following:

Data S1

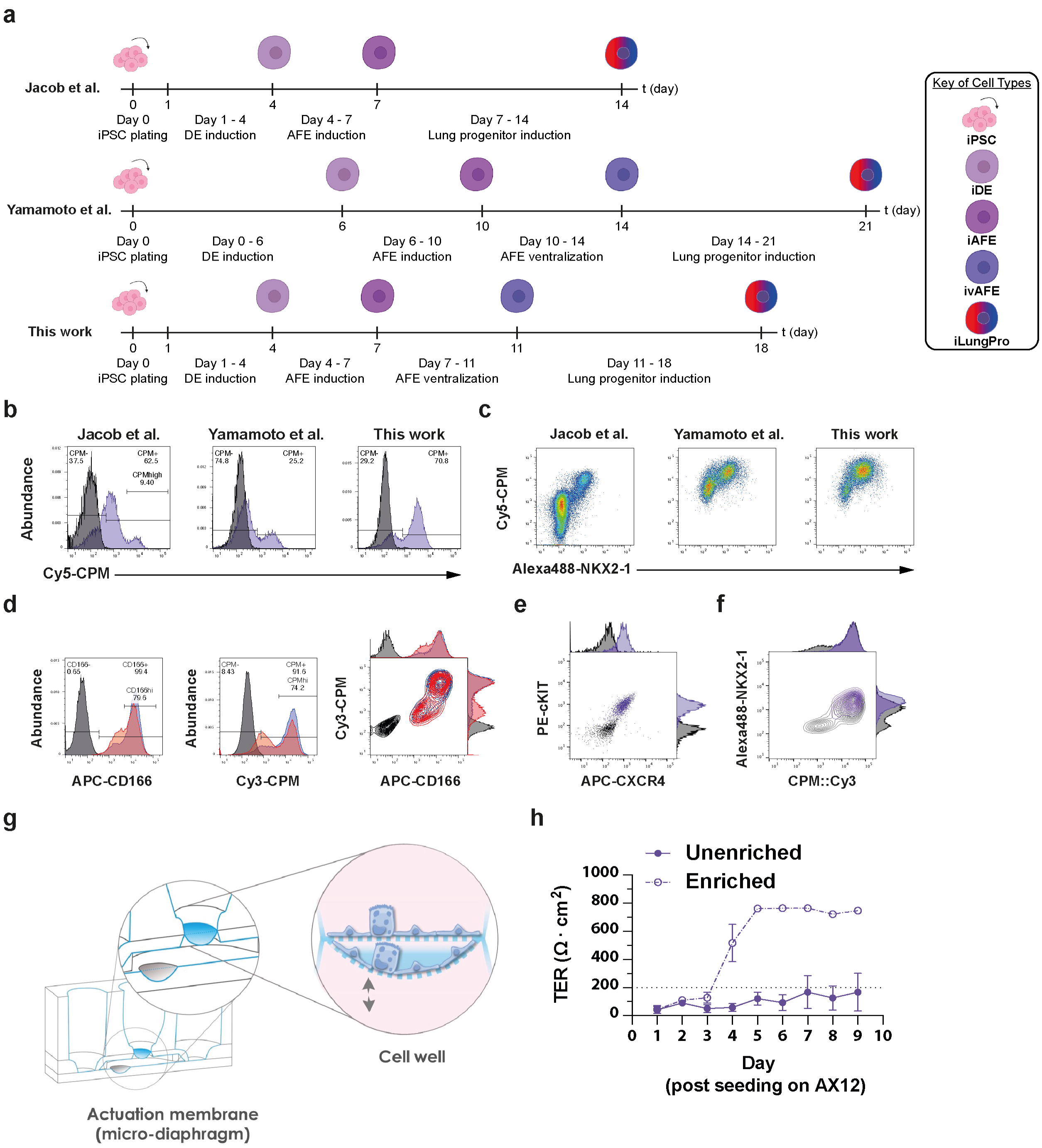

Supplementary Fig. 1. iPSC differentiation into iLungPro and iAT2&iAT1.

a, Schematic diagram comparing iLungPro differentiation protocols from Jacob et al. 2017, Yamamoto et al. 2017 (*23, 25*) and this work. **b**, Expression of CPM in iLungPro using different differentiation protocol, signal measured by flow cytometry, representative plot showing isotype (grey) and stained (purple) samples using unit area for y-axis. **c**, Expression of CPM and NKX2-1 in iLungPro using different differentiation protocol, signal measured by flow cytometry; n = 3 independent experiments. **d**, Expression of CD166 and CPM in iLungPro using different cell splitting ratio, signal measured by flow cytometry, representative plot

showing isotype (grey), 1:1 split (red) and 1:3 split (blue) samples using unit area for y-axis; n = 3 independent experiments. **e**, Expression of CXCR4 and cKit in iPSC-derived definitive endoderm measured by flow cytometry, representative plot showing isotype (grey) and stained (purple) samples; n = 3 independent experiments. **f**, Expression of CPM and NKX2-1 in iLungPro measured by flow cytometry, representative plot showing unsorted (grey) and CPM+ enriched (purple) samples; n = 3 independent experiments. **g**, Schematic diagram highlighting the 3D mechanical stretching function of AX12. **h**, TER quantification of iAE on AX12 up to 9 days post-seeding, using unenriched (solid line) or FACS-enriched (dotted line) iLungPro; mean ± s.d., n = 2 independent experiments. Created in BioRender. Luk, J. (2026) https://BioRender.com/wirsrvz

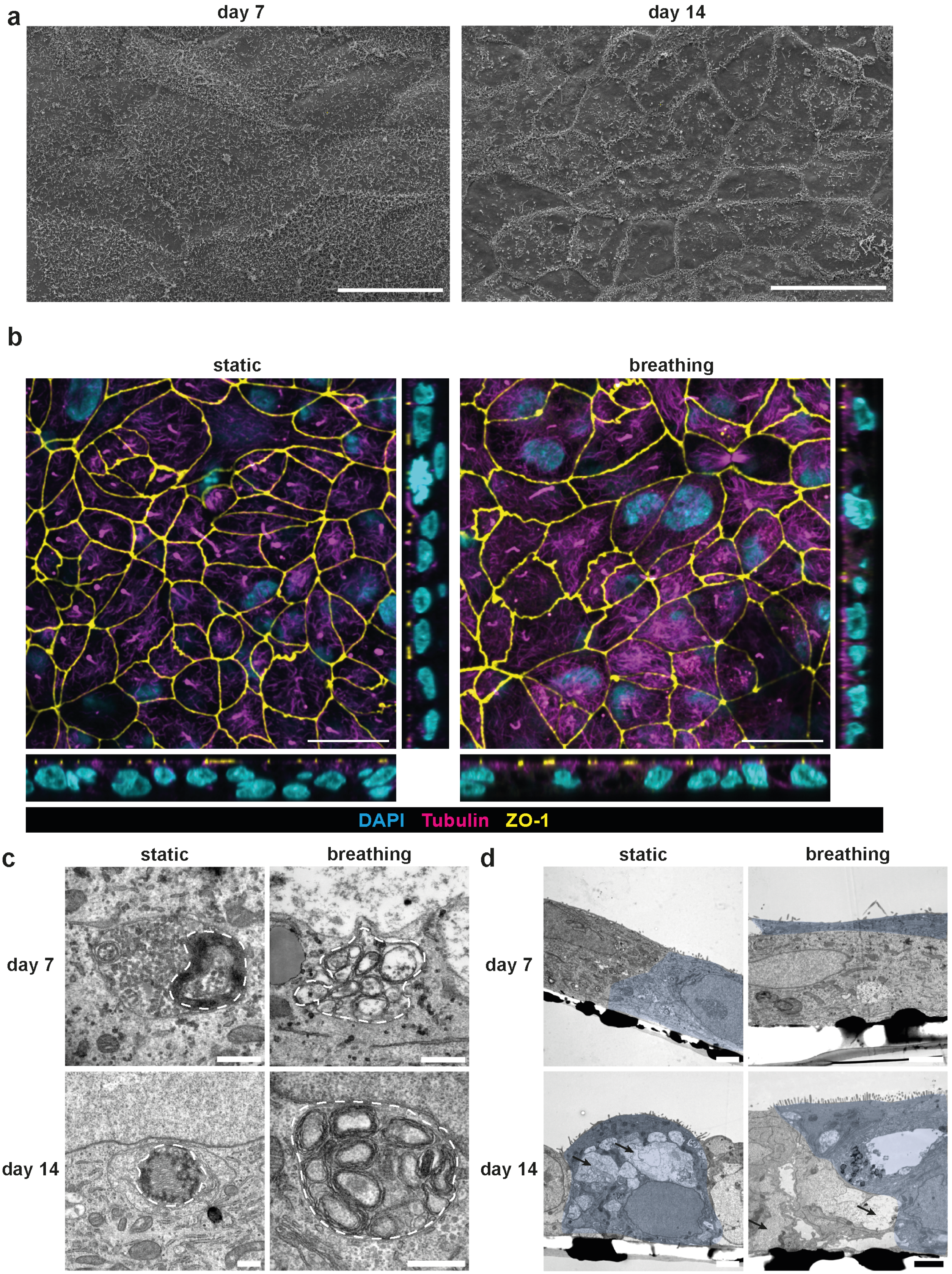

a
day 7
day 14
b
static
breathing
DAPI Tubulin ZO-1
c
static
breathing
day 7
day 14
d
static
breathing
day 7
day 14

Supplementary Fig. 2. Ultrastructural details of differentiated iAT2 and iAT1s.

a, SEM image of iAT2 and iAT1 differentiating on AX12 at 7 days post-seeding (left) and 14 days. Scalebar, 20 µm. **b**, Representative confocal images of orthogonal and planar view of the iAE on AX12 showing nuclei (cyan), acetylated Tubulin (magenta) and ZO-1 (yellow) at day 7 post-seeding, under static or breathing conditions. Scalebar, 20 µm. **c**, TEM images of lamellar bodies highlighted by dotted lines in iAT2 at day 7 and day 14 post-seeding, with or without mechanical stretch. Lamella bodies are depicted by dotted lines. Scalebar, 500 nm. **d**, TEM images of ciliated iAT2 and iAT1 cells highlighted in blue at day 7 and day 14 post-seeding, with or without mechanical stretch. Glycogen lake-like structures are indicated by arrows. Scalebar, 2 µm.

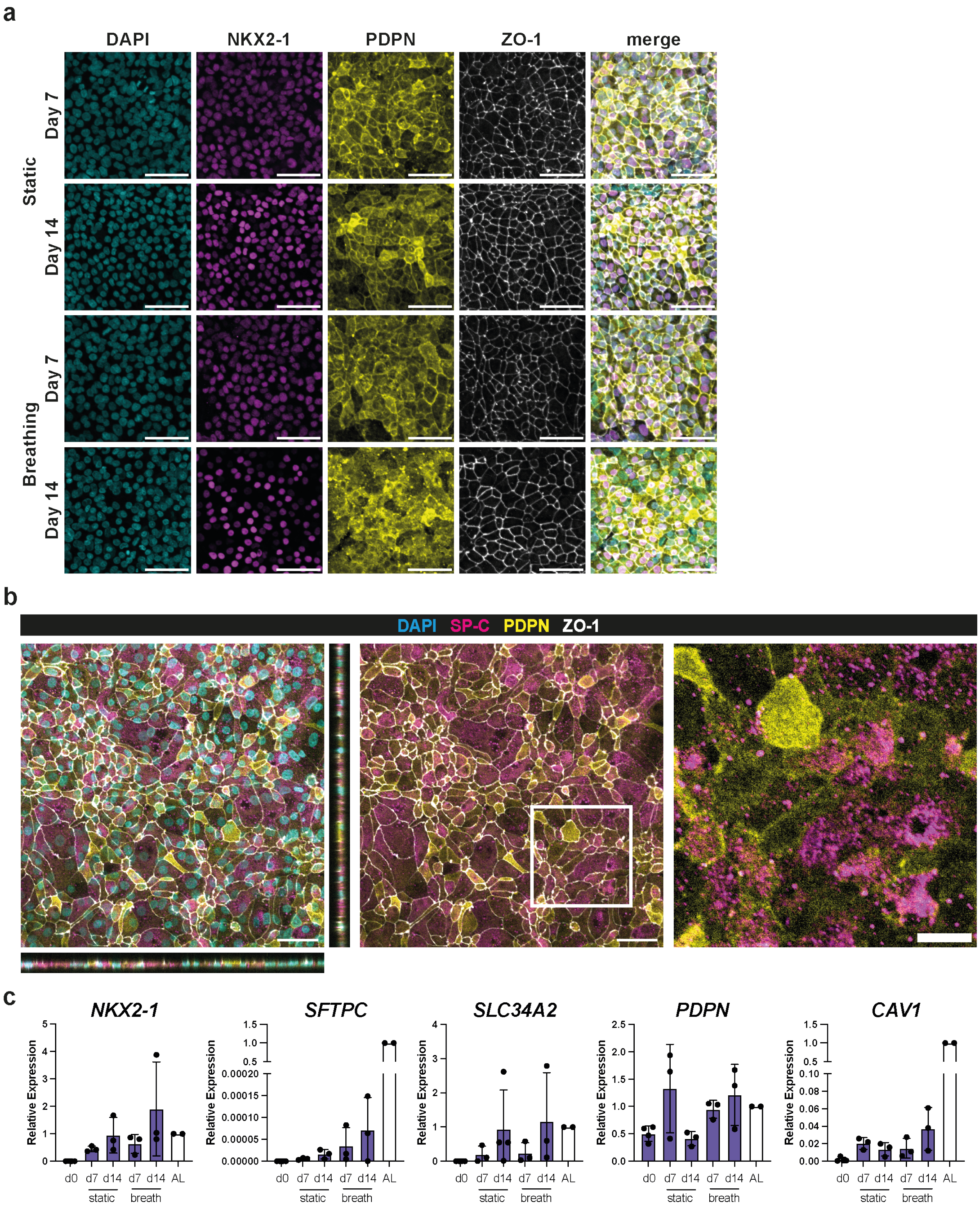

Supplementary Fig. 3. iAT2 and iAT1s in a Lung-on-chip microfluidic device are functional.
a, Representative confocal images of the iAE on AX12 showing nuclei (cyan), NKX2-1 (magenta), PDPN (yellow) and ZO-1 (white) at day 7 and day 14 post-seeding, under static or breathing conditions. Scalebar,

50 µm. **b**, Representative orthogonal view and magnified view of iAE on AX12 showing nuclei (cyan), mature SP-C (magenta), PDPN (yellow) and ZO-1 tight junctions (white) at day 14 post-seeding, under static conditions. Magnified view depicts square in the middle panel. Scale bar, 50 µm (orthogonal view) and 20 µm (magnified view). **c**, qPCR quantification of marker gene expression levels of iAT2 and iAT1, *NKX2-1*, *SFTPC*, *SLC34A2*, *PDPN* and *CAV1* in lung progenitors (d0), iAE cells at day 7 and day 14 post-seeding, under static or breathing conditions and primary adult lung (AL); mean ± s.d., n = 3 independent experiments.

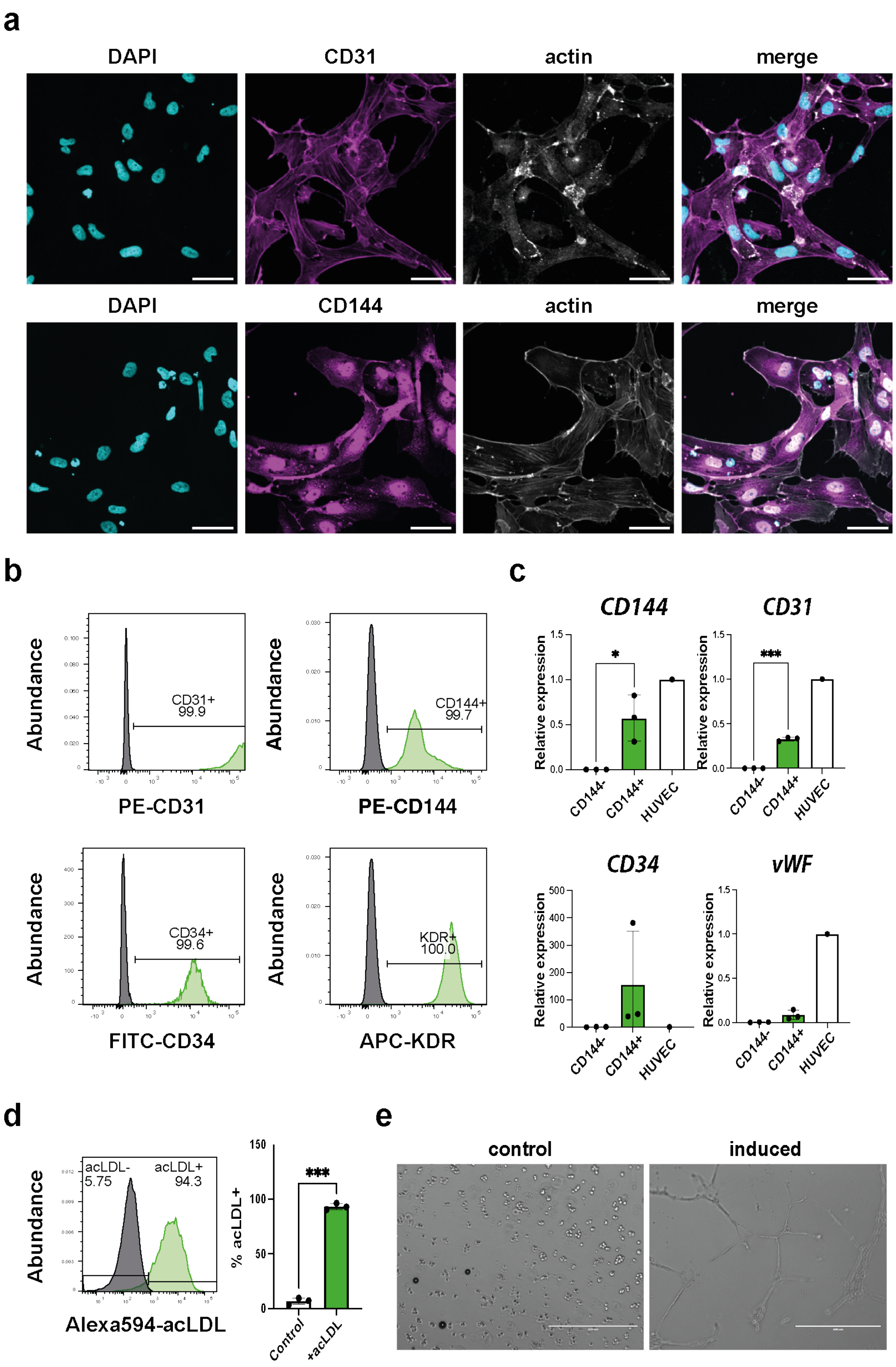

Supplementary Fig. 4. Differentiation and characterization of iVEC.
a, Representative confocal images of iVEC cultured on glass showing nuclei (cyan), CD31(top, magenta) or CD144 (bottom, magenta) and actin (white). Scalebar, 50 µm. **b**, Expression of CD31, CD144, CD34

and KDR in iVEC measured by flow cytometry, representative plots showing isotype (grey) and stained (green) samples; n = 3 independent experiments. **c**, Quantification of endothelial marker genes expression in iVEC by qPCR, graphs showing flowthrough cells of magnetic sorting (CD144-), magnetic enriched cells (CD144+) and HUVECs; mean ± s.d., n = 3 independent experiments, Student's t-test). **d**, Quantification of acLDL uptake by iVEC using flow cytometry, representative plots showing control (grey) and acLDL-treated (green) samples. Bar plot showing the quantification of three independent experiments; mean ± s.d., n = 3 independent experiments, Student's t-test. **e**, Representative brightfield images of iVEC undergoing angiogenesis, showing iVEC in control (Left) and induced (Right) conditions. Scalebar, 400 µm. p-value by unpaired t-test: * $p < 0.05$, *** $p < 0.001$.

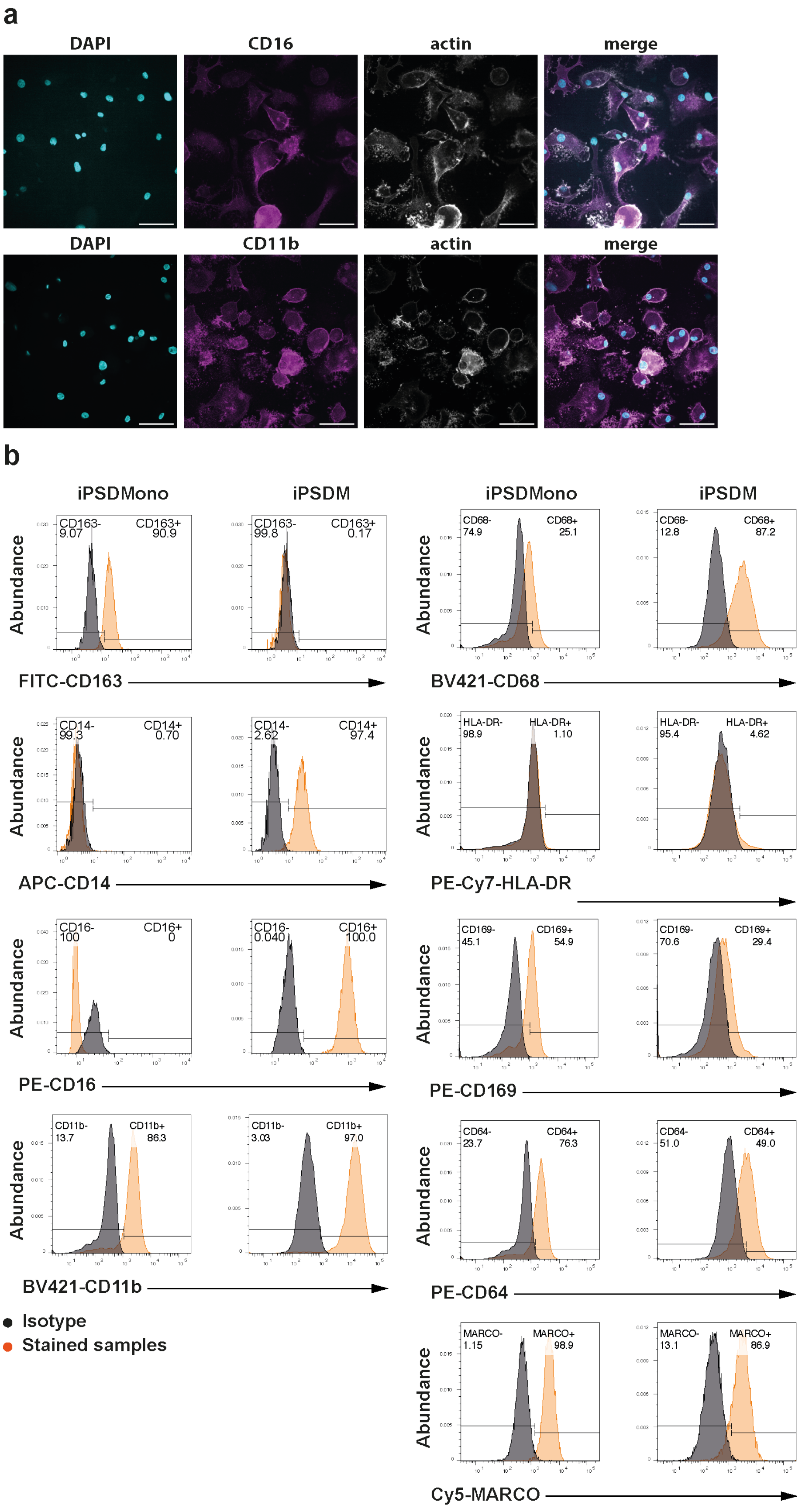
a
DAPI
CD16
actin
merge
DAPI
CD11b
actin
merge
b
iPSDMono
iPSDM
iPSDMono
iPSDM
Abundance
CD163- 9.07
CD163+ 90.9
CD163- 99.8
CD163+ 0.17
FITC-CD163
CD68- 74.9
CD68+ 25.1
CD68- 12.8
CD68+ 87.2
BV421-CD68
CD14- 99.3
CD14+ 0.70
CD14- 2.62
CD14+ 97.4
APC-CD14
HLA-DR- 98.9
HLA-DR+ 1.10
HLA-DR- 95.4
HLA-DR+ 4.62
PE-Cy7-HLA-DR
CD16- 100
CD16+ 0
CD16- 0.040
CD16+ 100.0
PE-CD16
CD169- 45.1
CD169+ 54.9
CD169- 70.6
CD169+ 29.4
PE-CD169
CD11b- 13.7
CD11b+ 86.3
CD11b- 3.03
CD11b+ 97.0
BV421-CD11b
CD64- 23.7
CD64+ 76.3
CD64- 51.0
CD64+ 49.0
PE-CD64
MARCO- 1.15
MARCO+ 98.9
MARCO- 13.1
MARCO+ 86.9
Cy5-MARCO
Isotype
Stained samples

Supplementary Fig. 5. Differentiation and characterization of iPSDM.

a, Representative confocal images of iPSDM cultured on glass showing nuclei (cyan), CD16 or CD11b (magenta) and actin (white). Scale bar, 50 µm. **b**, Expression of CD163, CD14, CD16, CD11b, CD68, HLA-DR, CD169, CD64 and MARCO in iPSDMono and iPSDM measured by flow cytometry, representative plots showing isotype (grey) and stained (orange) samples; n = 3 independent experiments.

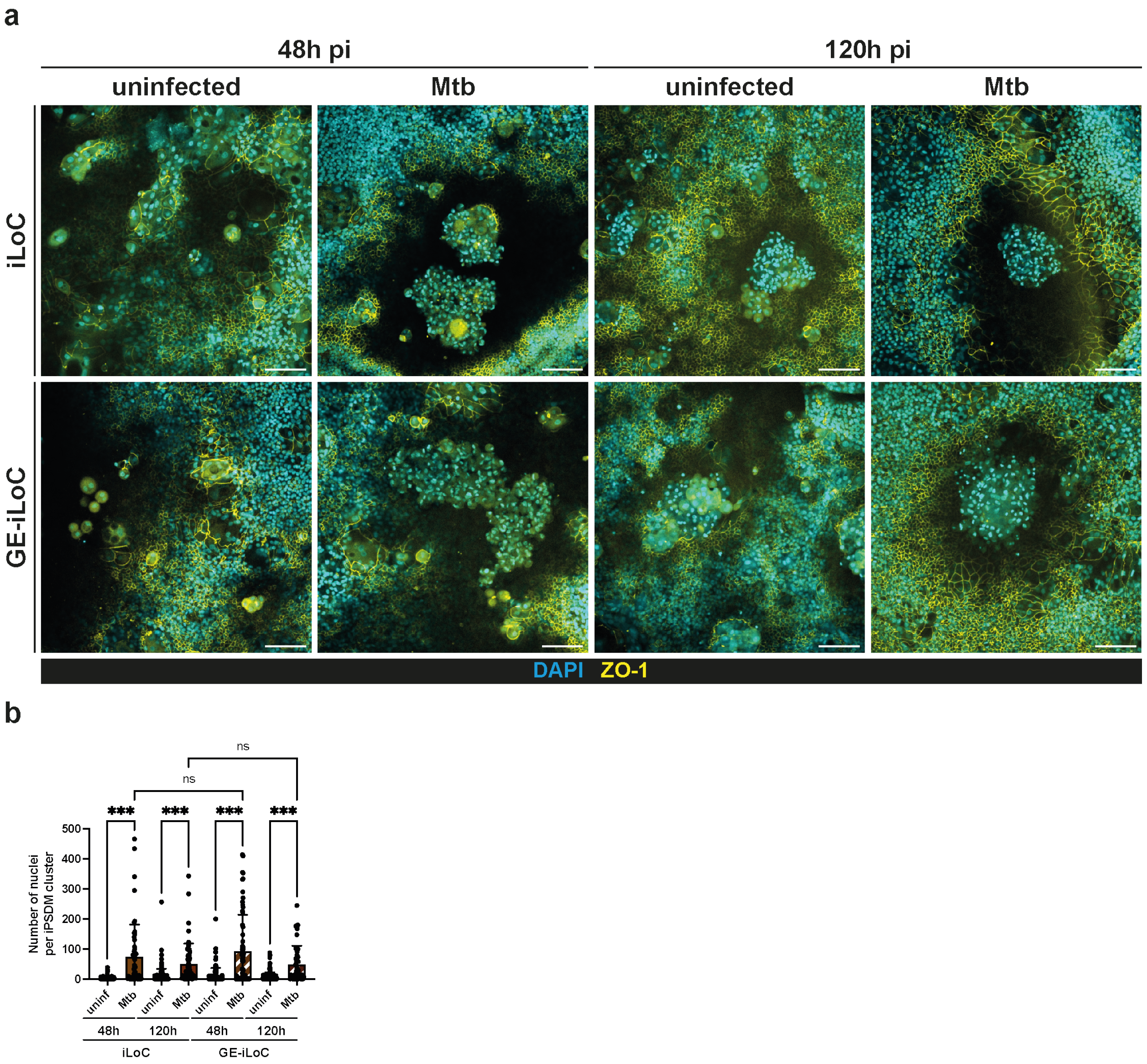

Supplementary Fig. 6. Macrophage clustering in the WT-iLoC and *ATG14KO* GE-iLoC before and after infection with *M. tuberculosis*

a. Representative confocal images of iPSDM in iLoC or GE-iLoC, image showing nuclei (cyan) and ZO-1 (yellow) under uninfected and infected conditions at 48 h and 120 h pi. Scalebar, 100 µm. **b,** Quantification of nuclei per iPSDM cluster in iLoC or GE-iLoC under uninfected and infected conditions at 48 h and 120 h pi. p-value: ns $P \geq 0.05$, *** $P < 0.001$.

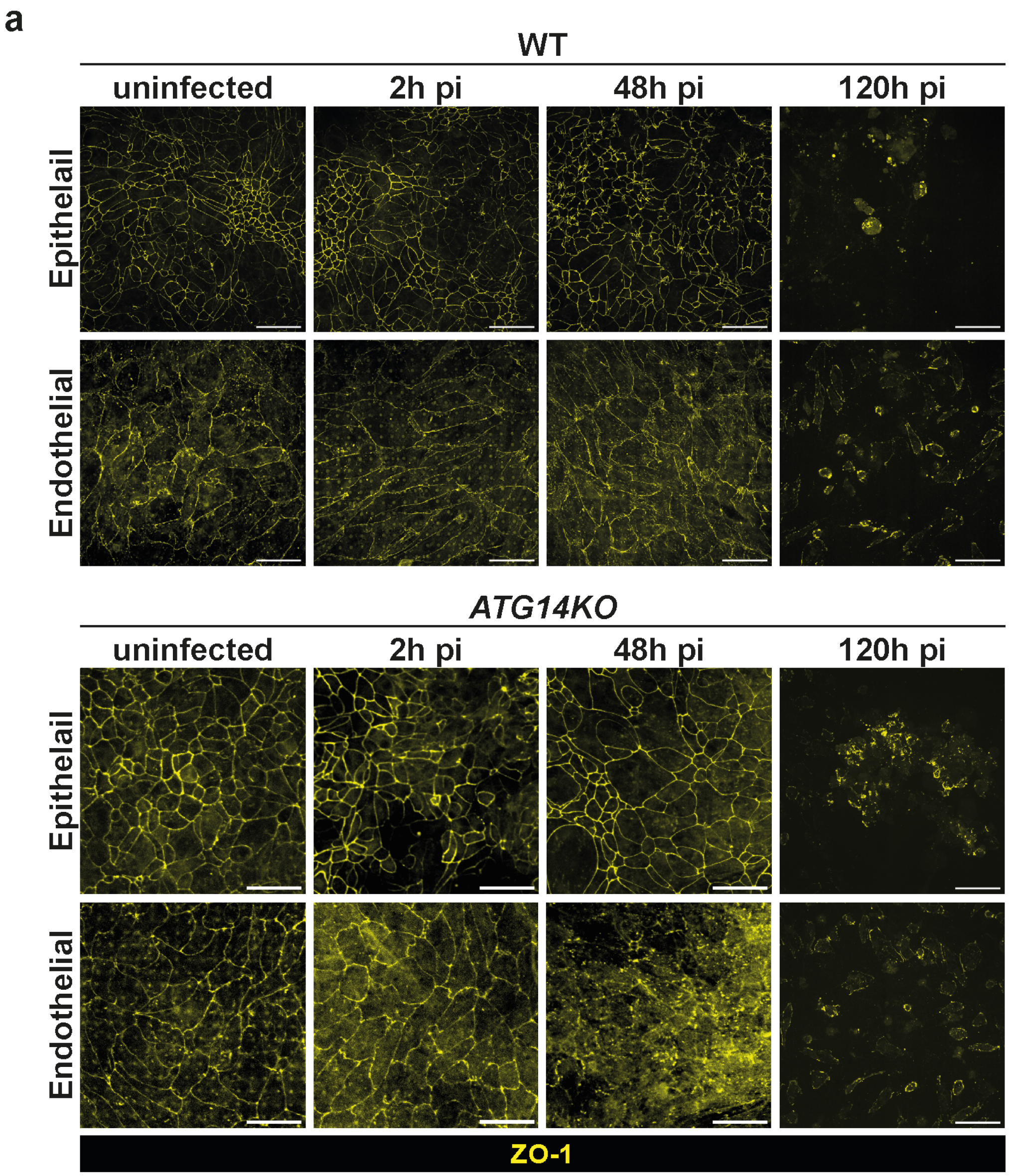

Supplementary Fig. 7. Epithelial and Endothelial barrier integrity in the WT-iLoC and *ATG14KO* GE-iLoC before and after infection with *M. tuberculosis*

a, Representative confocal images of tight junctions of epithelial and endothelial monolayer in iLoC or GE-iLoC marked by ZO-1, images showing ZO-1 (yellow). Scalebar, 50 µm.

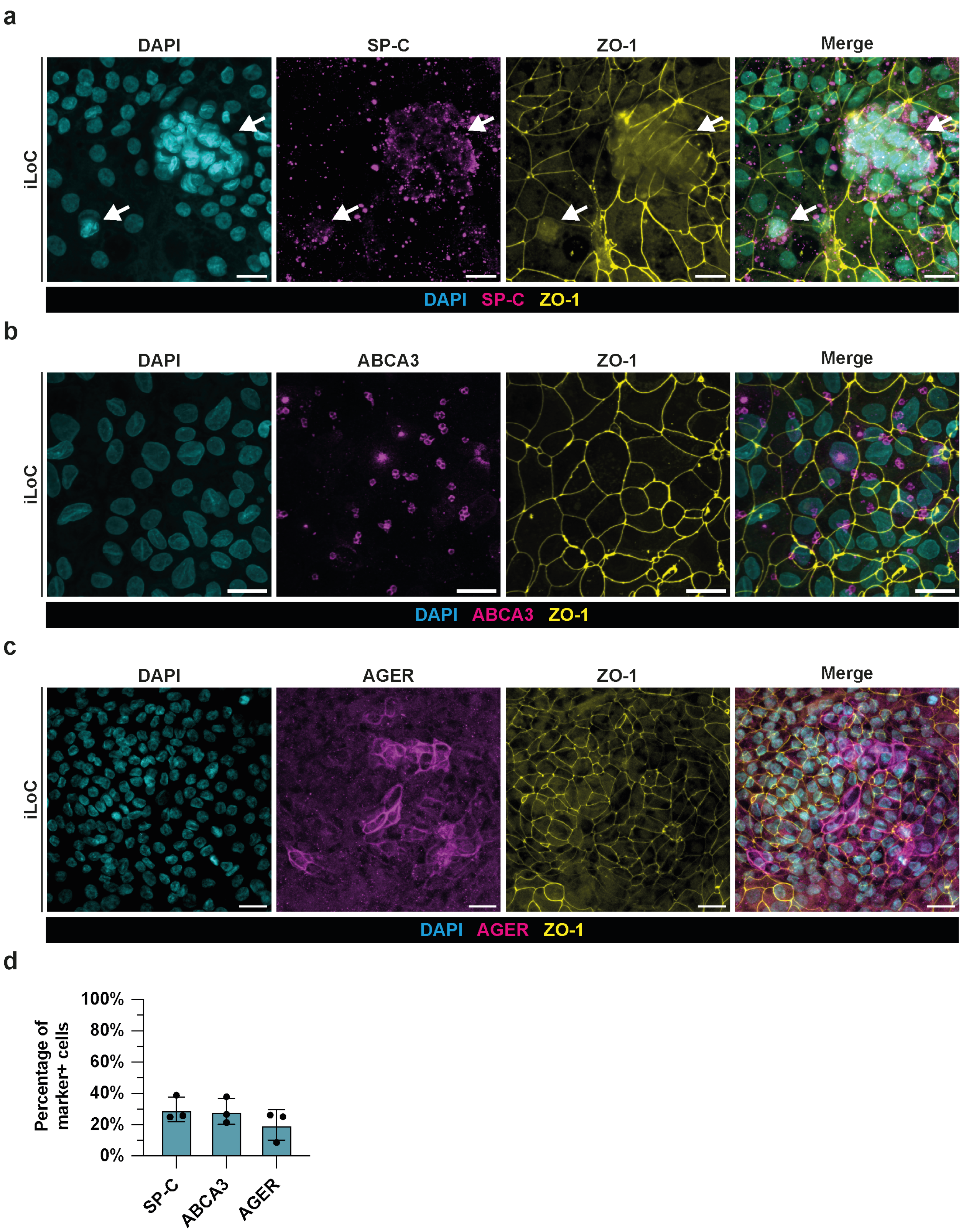

Supplementary Fig. 8. Expression of iAT2 and iAT1 markers of iLoC.

a, Representative confocal images of iLoC showing nuclei (cyan), SP-C (magenta) and ZO-1 (yellow) under static condition at day 21 of chip assembly, macrophages are depicted by arrows. Scalebar, 20 µm. **b**, Representative confocal images of iLoC showing nuclei (cyan), ABCA3 (magenta) and ZO-1 (yellow) under static condition at day 21 of chip assembly. Scalebar, 20 µm. **c**, Representative confocal images of iLoC showing nuclei (cyan), AGER (magenta) and ZO-1 (yellow) under static condition at day 21 of chip assembly. Scalebar, 20 µm. **d**, Quantification of SP-C, ABCA3 and AGER-expressing cells in under static condition at day 21 of chip assembly; mean ± s.d., n = 3 independent experiments.

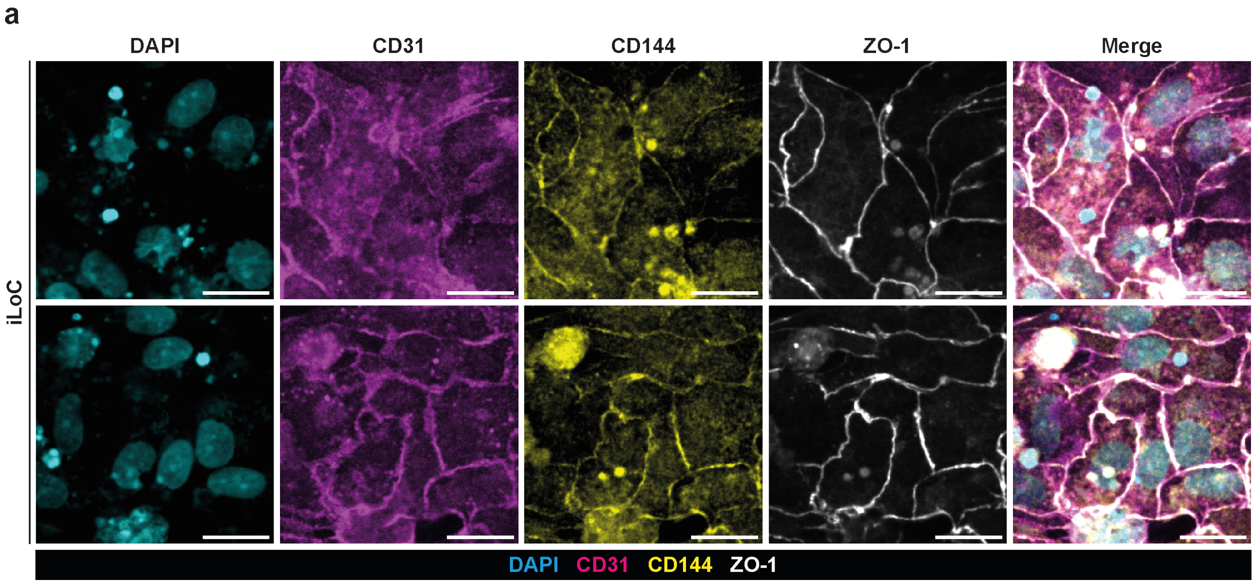

Supplementary Fig. 9. Expression of iVEC markers of iLoC.

a, Representative confocal images of iLoC showing nuclei (cyan), CD31 (magenta), CD144 (yellow) and ZO-1 (white) under static condition at day 21 of chip assembly. Scalebars: 20 μm.

a

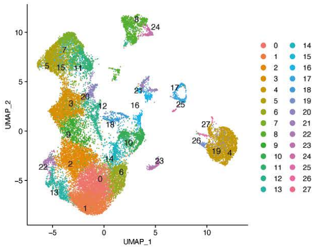

b

Annotated cell type	Clusters
iAT2	3, 9
Proliferating iAT2	10, 14, 18
iAT1	11, 12, 20
iVEC	8, 24
iPSDM	4, 19, 26, 27
Basal-like cell	5, 7, 15
Ciliated-like cell	17
Lung epithelial-like cell	0, 1, 2, 6, 13, 16, 21, 22, 23, 25

c

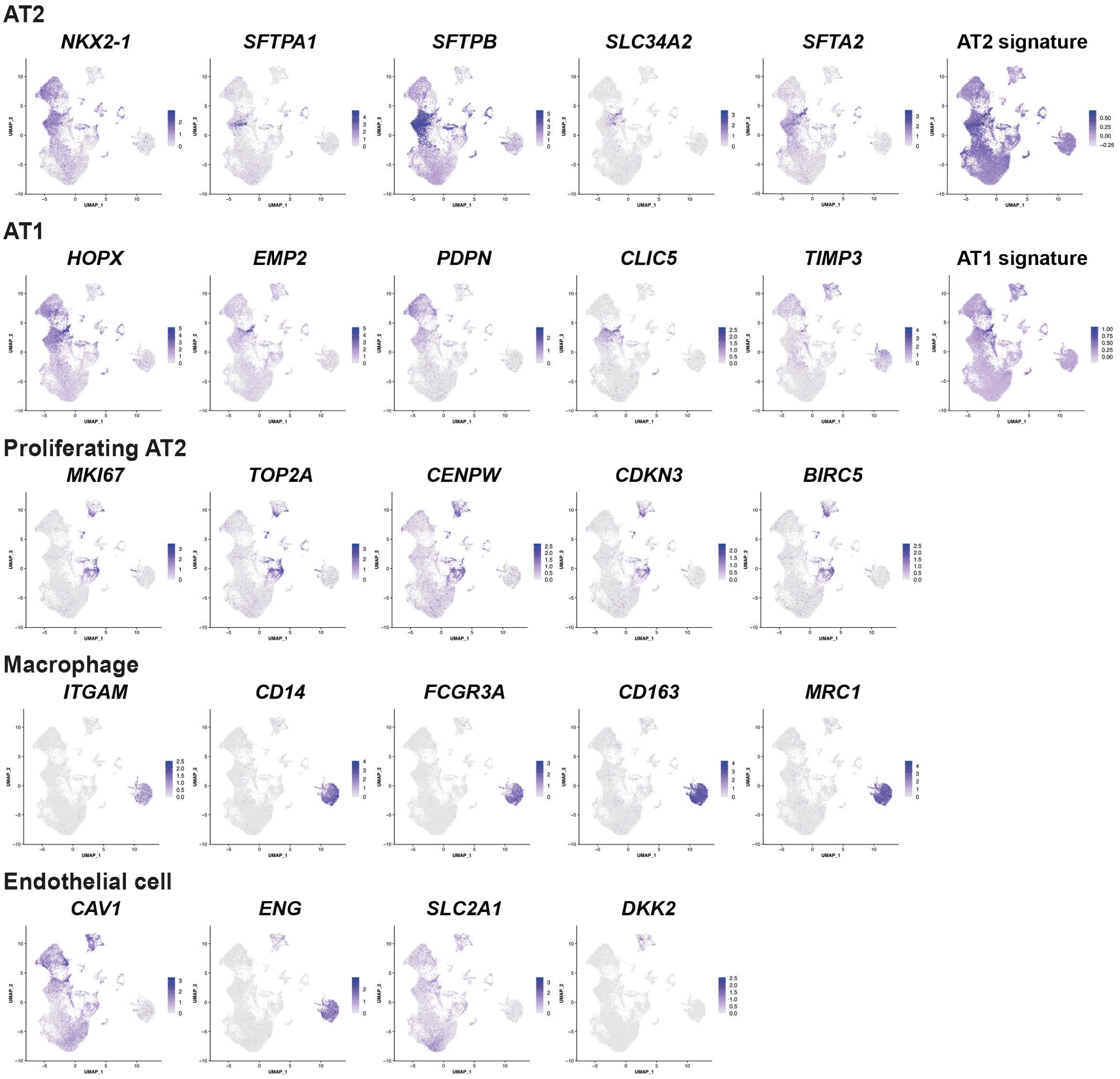

Supplementary Fig. 10. Clustering and cell annotation of iLoC single cell transcriptome analysis.

a, Uniform Manifold Approximation and Projection (UMAP) showing clusters at resolution 1 for the integrated analysis of 26907 iAM or iLoC-derived cells in the presence or absence of mechanical stretch. (N=1, n=1 per group). **b**, Cell annotation of clusters formed in a. **c**, Feature plots showing the expression of AT2 marker genes, *NKX2-1*, *SFTPA1*, *SFTPB*, *SLC34A2*, *SFTA2*, AT2 signature described in Burgess et al. 2024 (*21*); AT1 marker genes, *HOPX*, *EMP2*, *PDPN*, *CLIC5, TIMP3,* AT1 signature described in Burgess et al. 2024 (*21*); proliferating AT2 marker genes, *MKI67*, *TOP2A*, *CENPW*, *CDKN3*, *BIRC5*; macrophage marker genes, *ITGAM*, *CD14*, *FCGR3A*, *CD163*, *MRC1*; and endothelial cell marker genes, *CAV1*, *ENG*, *SLC2A1* and *DKK2* as shown in **Fig. 4c**.

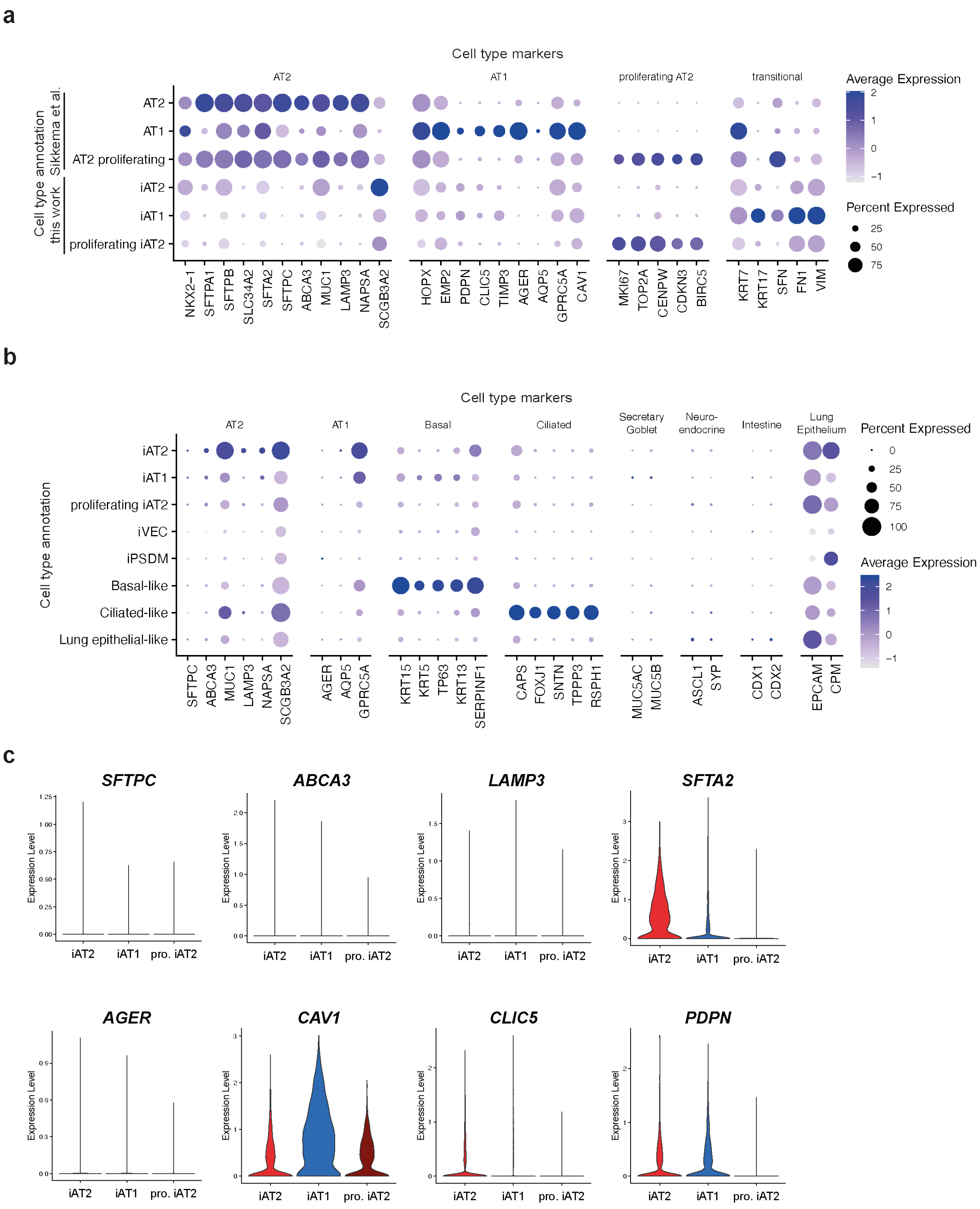

Supplementary Fig. 11. Expression tissue and cell type-specific markers of iLoC.

a, Dot plot representation of the expression of AT2, AT1, proliferating AT2 and transitional marker genes across AT2, AT1, AT2 proliferating clusters of Sikkema et al. 2023 (*46*) and annotated iAT2, iAT1 and pro. iAT2 clusters of AX12-derived cells from this study. **b**, Dot plot representation of the expression of additional AT2, AT1, basal cell, ciliated cell, secretary/goblet cell, neuroendocrine, intestinal, and lung epithelial cell gene markers across annotated cell types. **c**, Violin plots of the expression of AT2 marker genes, *SFTPC*, *ABCA3*, *LAMP3*, *SFTA2*; and AT1 marker genes, *AGER*, *CAV1*, *CLIC5*, *PDPN* in annotated iAT2, iAT1 and pro. iAT2 clusters of AX12-derived cells from this study.

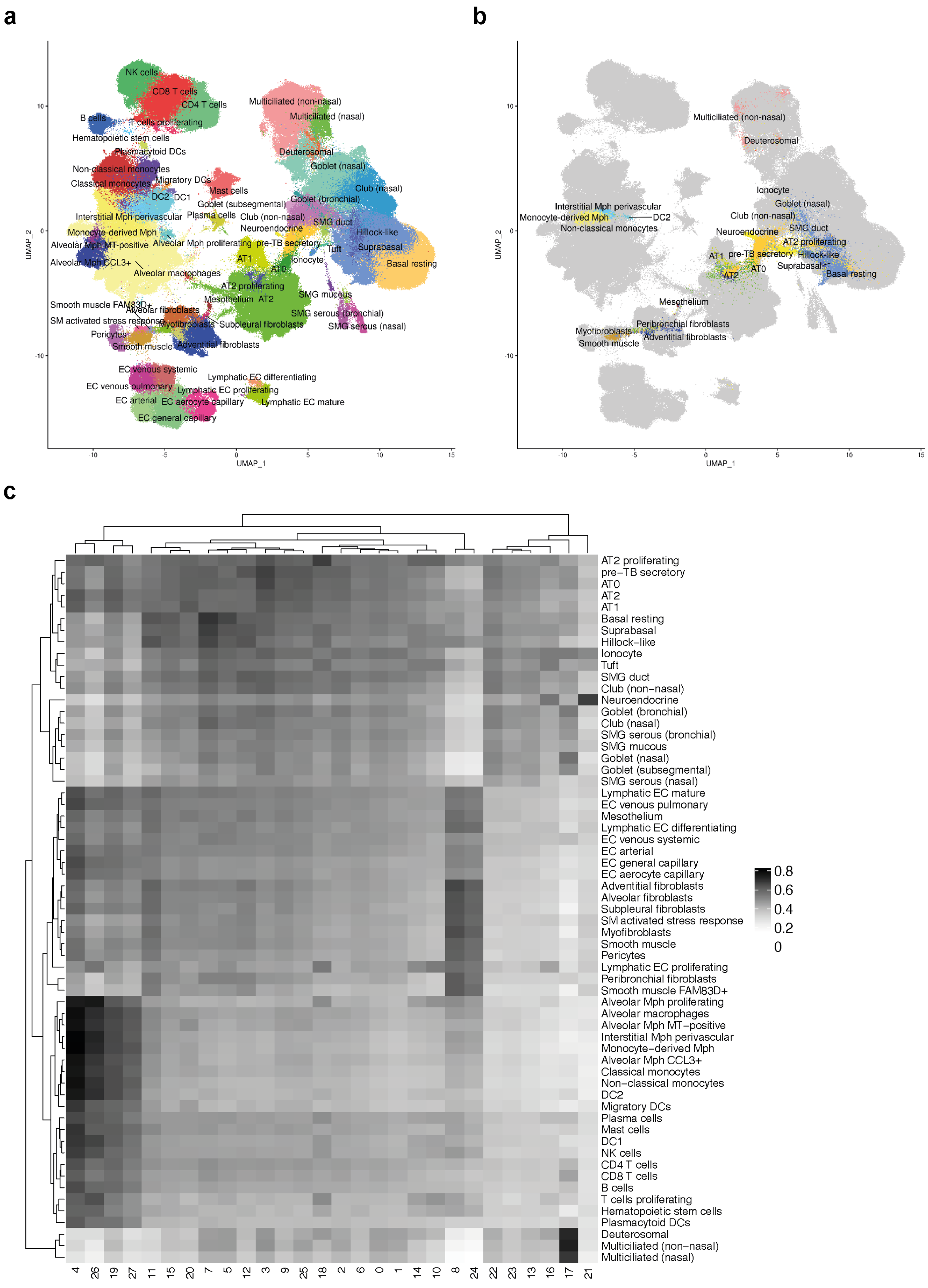

Supplementary Fig. 12. Mapping of iLoC single cell transcriptome to reference dataset.

a, UMAP visualization of cell identity annotation of the integrated healthy Human Lung Cell Atlas (HLCA) reference dataset published by Sikkema et al. 2023 (*46*). **b**, UMAP projection of iAM or iLoC-derived cells in the presence or absence of mechanical stretch (query cells) onto the reference HLCA UMAP in **a**. **c**, Heatmap showing spearman correlation coefficients between reference HLCA cell types from Sikkema et al. 2023 (*46*) and AX12-derived cells (query clusters at resolution 1) from this study.

a

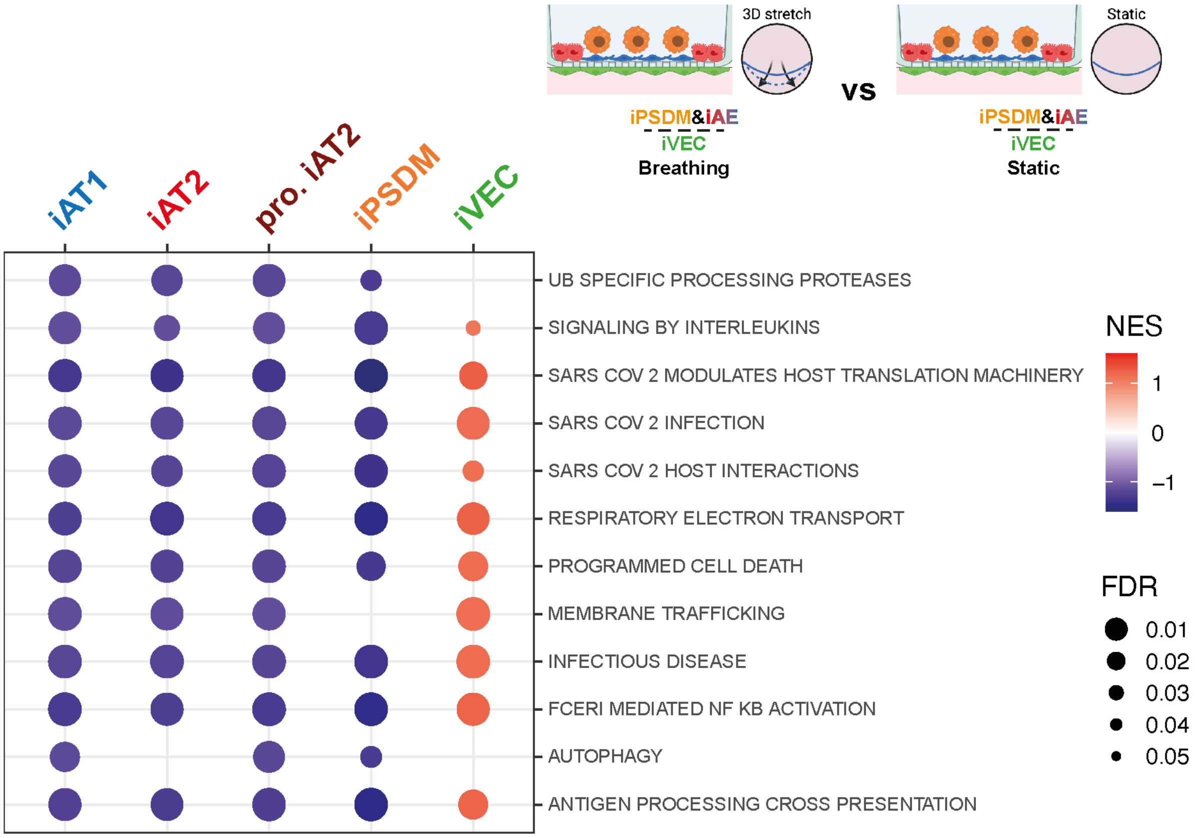

Supplementary Fig. 13. Pathway analysis of iLoC

a, Dot plot showing selected Reactome enriched pathways from GSEA analysis for differential expression analysis in iAT2, iAT1, pro. iAT2, iPSDM and iVEC cell types from iLoC under breathing and static condition (Breathing vs Static). Created in BioRender. Luk, J. (2026) https://BioRender.com/wirsrvz

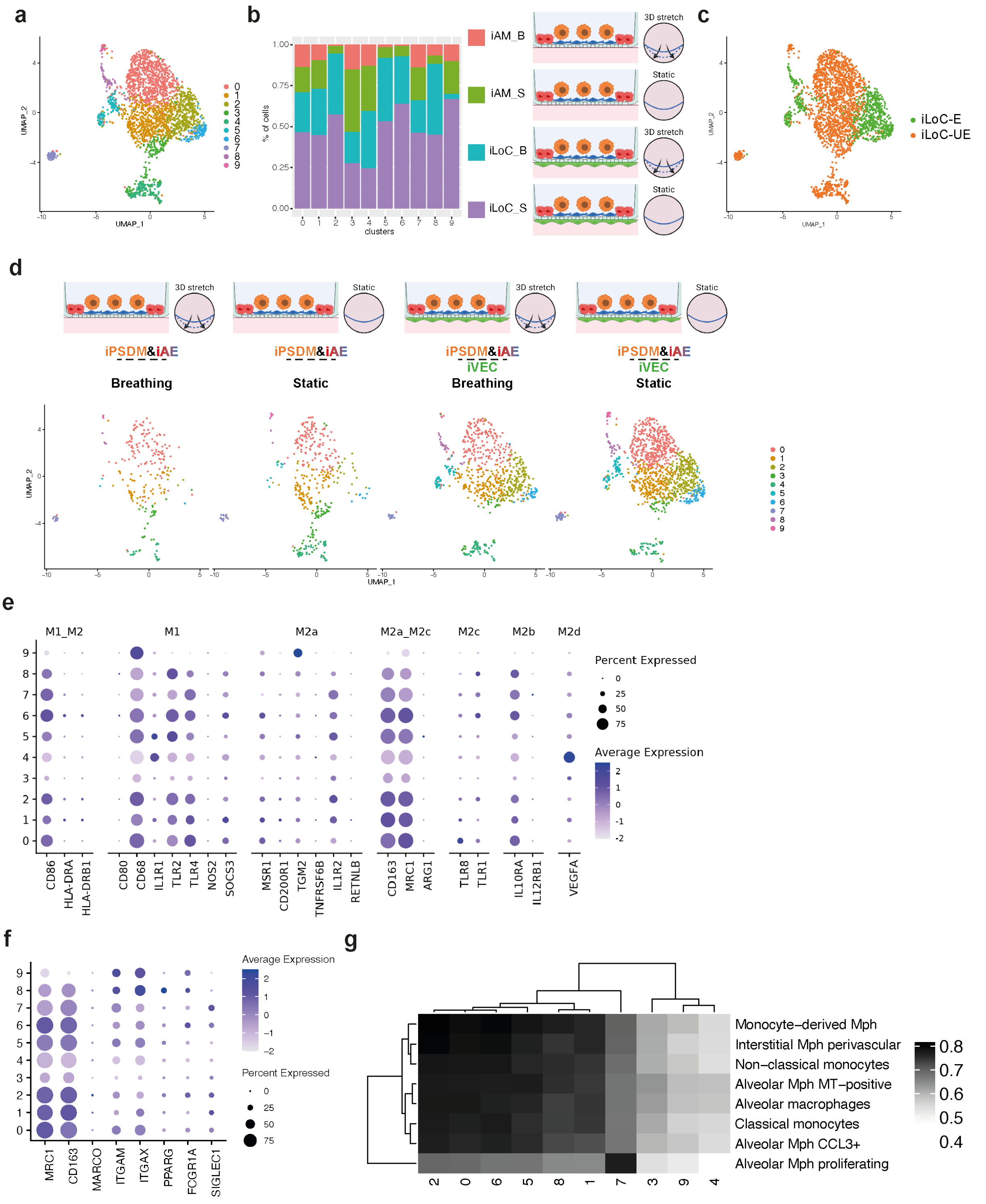

Supplementary Fig. 14. Clustering and subpopulation analysis of iPSDM on iLoC.

a, UMAP visualization of subclusters identified from iPSDM population from iLoC. **b**, Cell proportions of iPSDM subclusters in each individual sample from **a**. **c**, UMAP visualization of subclusters identified from iPSDM population from iLoC, highlighting iLoC enriched (green) and unenriched (orange) subclusters. **d**, UMAP visualization of iPSDM subclusters from **a** split by experimental condition. **e**, Dot plot

representation of the expression of selected macrophage phenotype genes across all iPSDM identified subclusters. **f**, Dot plot representation of the expression of selected alveolar macrophage marker genes across all iPSDM identified subclusters. **g**, Heatmap showing spearman correlation coefficients between macrophage cell types from HLCA from Sikkema et al. 2023 (*46*) and iPSDM subclusters from this study. Created in BioRender. Luk, J. (2026) https://BioRender.com/wirsrvz

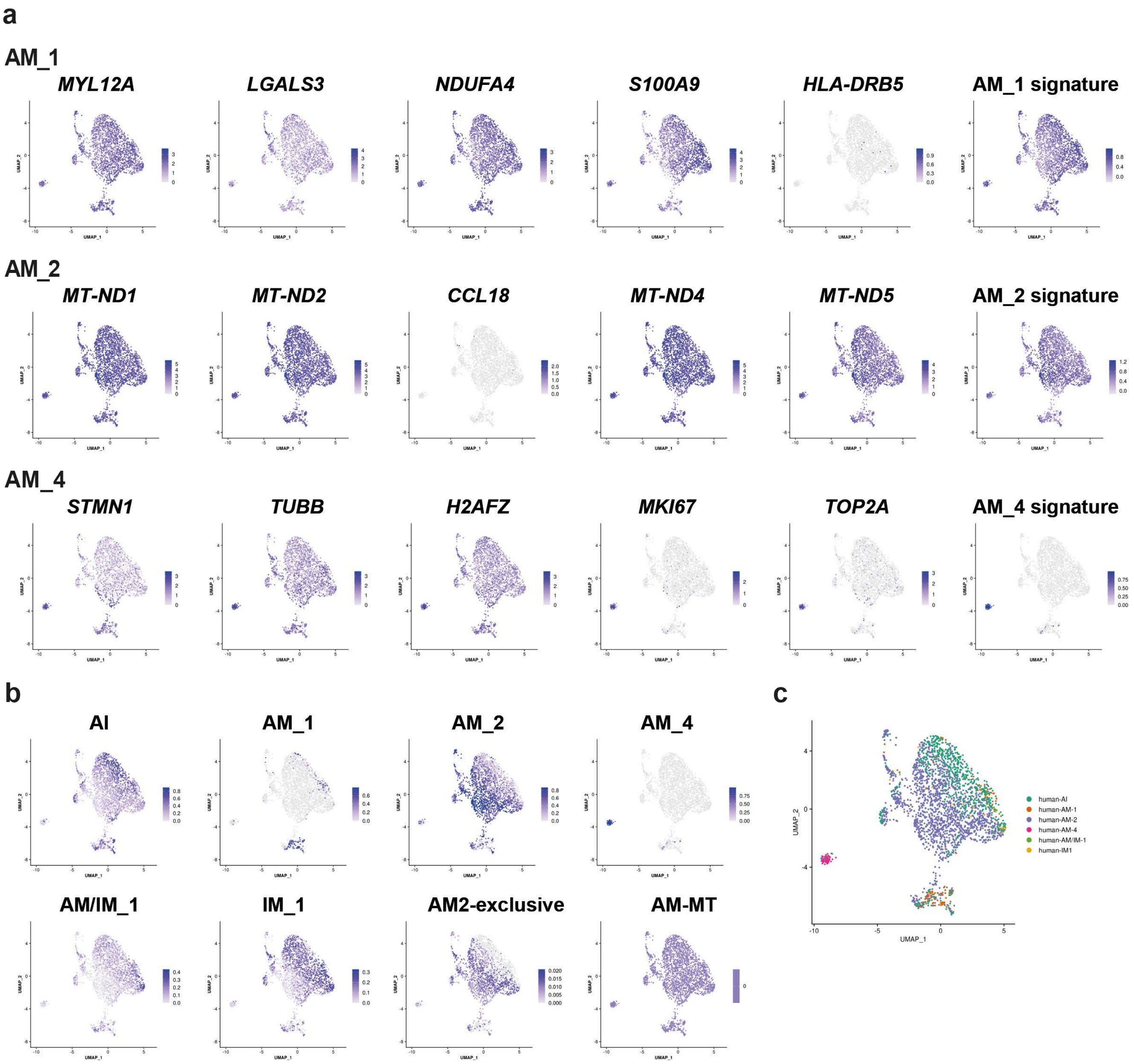

Supplementary Fig. 15. Subpopulation analysis of iPSDM on iLoC in reference to human lung. **a**, UMAP of iPSDM showing the expression of the top DEGs of alveolar macrophage subsets described in Pisu et al. 2021 (*48*). **b**, UMAP of iPSDM showing the predicted scores of cell identity from alveolar macrophage subsets described in Pisu et al. 2021(*48*). **c**, UMAP of iPSDM showing the predicted cell identity from alveolar macrophage subsets described in Pisu et al. 2021(*48*).

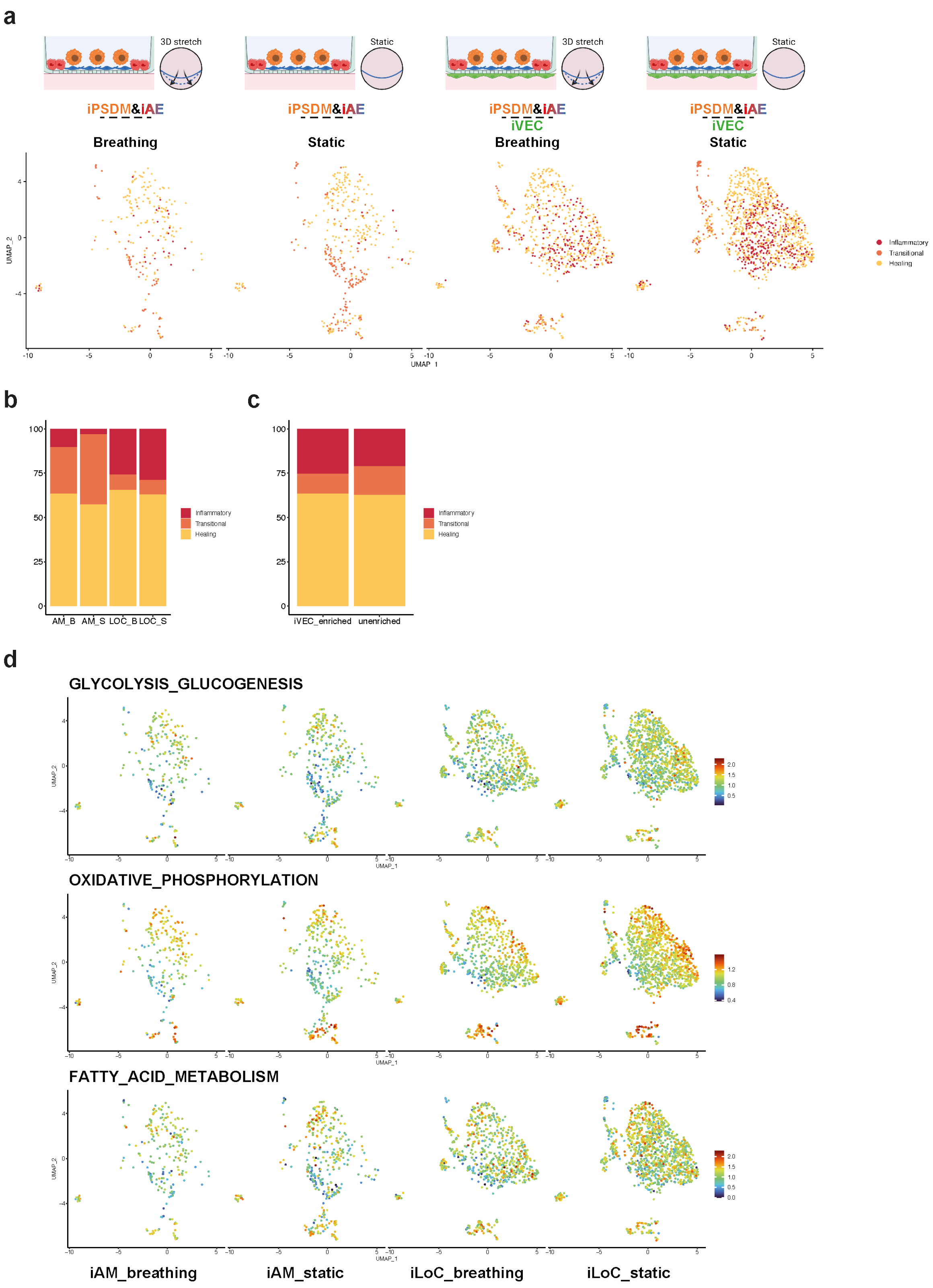

Supplementary Fig. 16. Macrophage polarity and metabolic analysis of iPSDM on iLoC using MACanalyzeR.

a, UMAP of iPSDM colored by MacPolarizeR and split by iLoC conditions. **b**, Quantification of macrophage phenotype in different iLoC conditions. **c**, Quantification of macrophage phenotype in iLoC-E and iLoC-UE iPSDM. **d**, PathAnalyzeR UMAP of iPSDM showing expression of glycolysis, gluconeogenesis, oxidative phosphorylation and fatty acid metabolism KEGG pathways. Created in BioRender. Luk, J. (2026) https://BioRender.com/wirsrvz

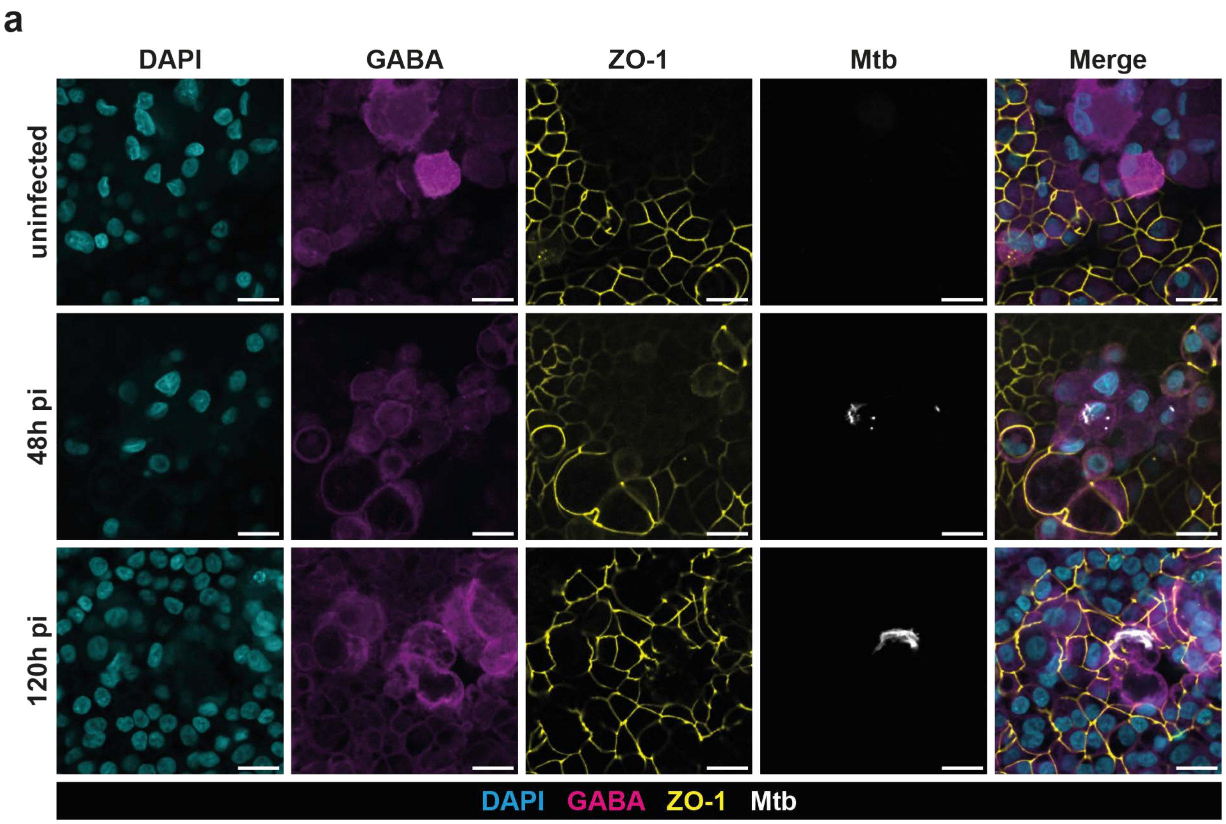

Supplementary Fig. 17. Expression of metabolic marker in iLoC.

a, Representative confocal images of uninfected and infected iLoC at 48 h and 120 h pi, showing nuclei (cyan), GABA (magenta), ZO-1 (yellow) and Mtb (white). Scalebar, 20 µm.

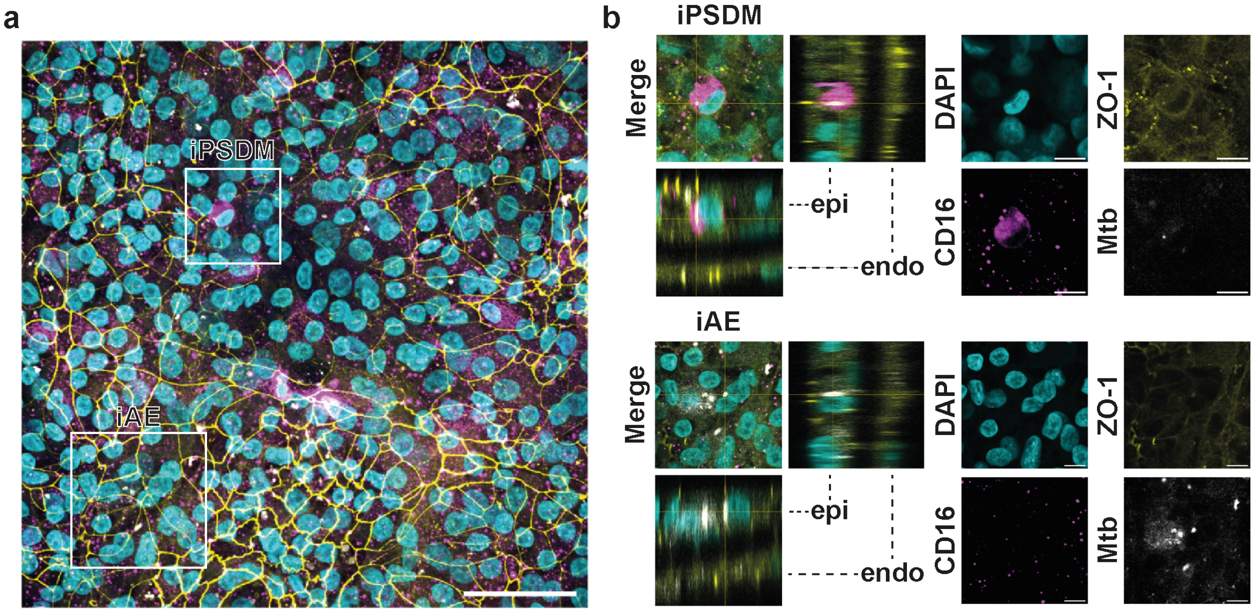

Supplementary Fig. 18. Cytokine profiling of WT-iLoC and *ATG14KO* GE-iLoC.

a, Representative confocal image of Mtb-infected iLoC depicting Mtb dwelling in iAE, and iPSDM at 2 h pi. Scalebar, 50 µm. **b**, Zoomed images from **a** showing: nuclei (cyan), CD16 (magenta), ZO-1 (yellow) and Mtb (white). Scalebar, 10 µm.

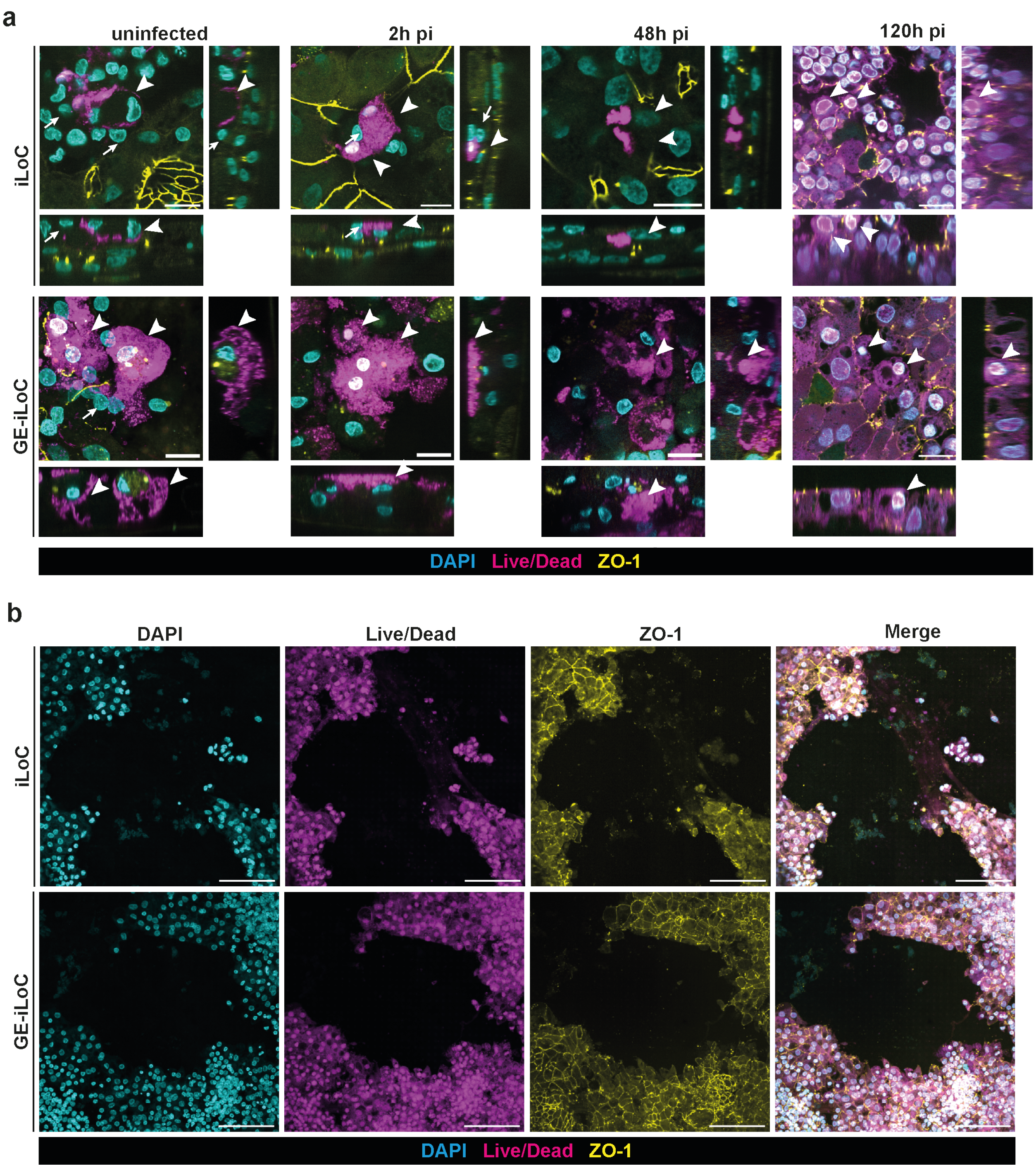

Supplementary Fig. 19 Cell death in the iLoC and *ATG14KO* GE-iLoC after infection with *M. tuberculosis*

a, Representative confocal images of cell death in iPSDM in iLoC or GE-iLoC indicated by Live/Dead Orange, images showing nuclei (cyan), Live/Dead Orange (magenta) and ZO-1 (yellow) under uninfected condition, 2 h, 48 h and 120 h pi. Live and dead cells were depicted by arrow and arrowhead, respectively. Scalebar, 20 μm. **b**, Representative images of cell death in iPSDM in iLoC or GE-iLoC indicated by

Live/Dead Orange, images showing nuclei (cyan), Live/Dead Orange (magenta) and ZO-1 (yellow) at 120 h pi. Scalebar, 100 μm.

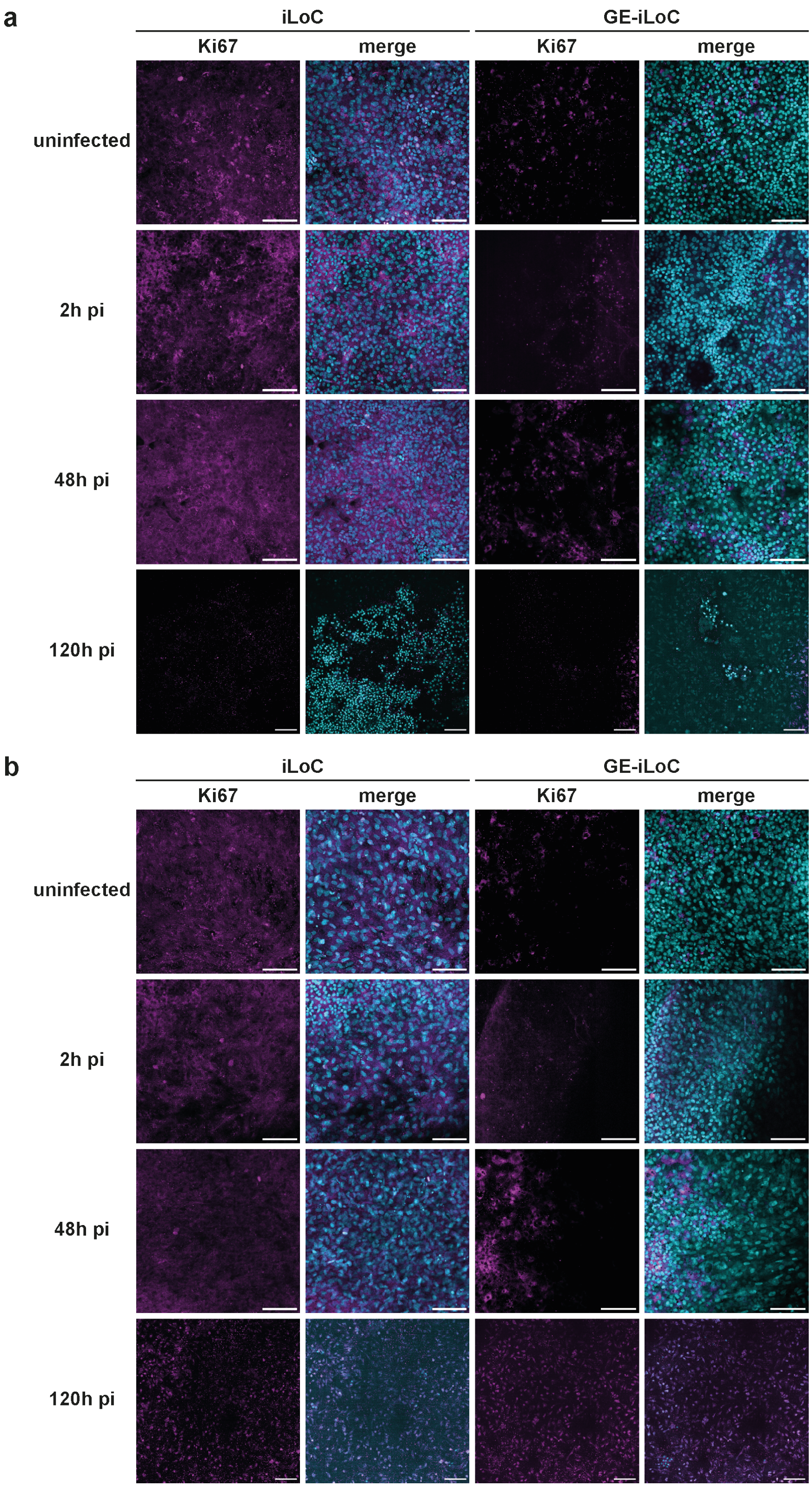

Supplementary Fig. 20. Cell proliferation in the WT-iLoC and *ATG14KO* GE-iLoC after infection with *M. tuberculosis*

a, Representative confocal images of cell proliferation in the epithelial side of iLoC or GE-iLoC indicated by Ki67 at different infection timepoints, images showing nuclei (cyan) and Ki67 (magenta) under uninfected condition, 2 h, 48 h and 120 h pi. Scalebar, 100 µm. **b**, Representative images of cell proliferation in the endothelial side of iLoC or GE-iLoC indicated by Ki67 at different infection timepoints, images showing nuclei (cyan) and Ki67 (magenta) under uninfected condition, 2 h, 48 h and 120 h pi. Scalebar, 100 µm.

Table S1.
List of reagents and equipment used in this work.

REAGENT or RESOURCE	SOURCE	IDENTIFIER
**Antibodies**		
CD184 (CXCR4) Monoclonal Antibody (12G5), PE, eBioscience	Thermo Fisher Scientific	Cat# 12-9999-42, RRID:AB_10669164
APC anti-human CD117 (c-kit)	BioLegend	Cat# 313206 (also 313205), RRID:AB_314985
Anti CPM, Monoclonal Antibody (WK)	FUJIFILM Wako Shibayagi	Cat# 014-27501, RRID:AB_2801482
Recombinant Anti-TTF1 antibody [SP141]	Abcam	Cat# ab227652, RRID:AB_3096124
Anti-Mature-SP-C	Seven Hills Bioreagents	Cat# WRAB-76694, RRID:AB_2938817
Purified anti-human Podoplanin	BioLegend	Cat# 337001 (also 337002), RRID:AB_1595616
ZO-1 Monoclonal Antibody (ZO1-1A12), Alexa Fluor™ 488	Thermo Fisher Scientific	Cat# MA3-39100-A488, RRID:AB_2663169
ZO-1 Monoclonal Antibody (ZO1-1A12), Alexa Fluor™ 647	Thermo Fisher Scientific	Cat# MA3-39100-A647, RRID:AB_2663167
APC anti-human CD166	BioLegend	Cat# 343906 (also 343905), RRID:AB_2832670
PE anti-human CD26	BioLegend	Cat# 302706 (also 302705), RRID:AB_314290
PerCP/Cyanine5.5 anti-human CD47	BioLegend	Cat# 323110 (also 323109), RRID:AB_940461
CD31 Polyclonal Antibody	Thermo Fisher Scientific	Cat# PA5-16301, RRID:AB_10981955
CD144 (VE-cadherin) Monoclonal Antibody (16B1), eBioscience	Thermo Fisher Scientific	Cat# 14-1449-82, RRID:AB_467495
PE anti-human CD31	BioLegend	Cat# 303105 (also 303106), RRID:AB_314331
Mouse Anti-CD34 Monoclonal Antibody, FITC Conjugated, Clone 581	BD Biosciences	Cat# 555821, RRID:AB_396150
Mouse Anti-Human CD144 Monoclonal Antibody, PE Conjugated, Clone 55-7H1	BD Biosciences	Cat# 560410, RRID:AB_1645502
APC anti-human CD309 (VEGFR2)	BioLegend	Cat# 393006 (also 393005), RRID:AB_2750143
Recombinant Anti-CD16 antibody [EPR22409-124]	Abcam	Cat# ab246222, RRID:AB_2910166
CD11b Monoclonal Antibody (M1/70), eBioscience	Thermo Fisher Scientific	Cat# 14-0112-82, RRID:AB_467108

Mouse Anti-Human CD163 Monoclonal Antibody, FITC Conjugated, Clone GHI/61	BD Biosciences	Cat# 563697, RRID:AB_2738379
Mouse Anti-CD14 Monoclonal Antibody, Allophycocyanin Conjugated, Clone M5E2	BD Biosciences	Cat# 555399, RRID:AB_398596
Mouse Anti-Human CD16 Monoclonal Antibody, PE Conjugated, Clone B73.1	BD Biosciences	Cat# 561313, RRID:AB_10643606
Alexa Fluor® 594 Anti-CD16 antibody [SP175]	Abcam	Cat# ab310761, AB_3096129
Anti-Ki67 antibody	Abcam	Cat# ab15580, RRID:AB_443209
Anti-Human Von Willebrand Factor Antibody	Agilent	Cat# A0082 (also A008202-5, A008202-2), RRID:AB_2315602
Anti-ABCA3 antibody	Seven Hills Bioreagents	Cat# WRAB-70565, RRID:AB_3146162
Human/Mouse/Rat RAGE/AGER Antibody	R and D Systems	Cat# AF1145, RRID:AB_354628
Rabbit Anti-GABA Antibody, Unconjugated	Sigma-Aldrich	Cat# A2052, RRID:AB_477652
Goat anti-Mouse IgG (H+L) Cross-Adsorbed Secondary Antibody, Cyanine3	Thermo Fisher Scientific	Cat# A10521, RRID:AB_2534030
Goat Anti-Rabbit IgG (H+L) Antibody, Alexa Fluor 488 Conjugated	Thermo Fisher Scientific	Cat# A-11008 (also A11008), RRID:AB_143165
Goat anti-Rabbit IgG (H+L) Cross-Adsorbed Secondary Antibody, Cyanine3	Thermo Fisher Scientific	Cat# A10520, RRID:AB_10563288
Goat anti-Rabbit IgG (H+L) Cross-Adsorbed Secondary Antibody, Cyanine5	Thermo Fisher Scientific	Cat# A10523, RRID:AB_2534032
PerCP/Cy5.5 Mouse IgG1, κ Isotype Ctrl Antibody	BioLegend	Cat# 400150, RRID:AB_893664
Mouse IgG1, kappa Isotype Control, FITC Conjugated, Clone MOPC-21	BD Biosciences	Cat# 555748, RRID:AB_396090
PE Mouse IgG1, κ Isotype Ctrl Antibody	BioLegend	Cat# 400112 (also 400111), RRID:AB_2847829
PE Mouse IgG2a, κ Isotype Ctrl Antibody	BioLegend	Cat# 400212, RRID:AB_326460
PE Mouse IgG1, κ Isotype Control	BD Biosciences	Cat# 555749, RRID:AB_396091
APC Mouse IgG1, κ Isotype Ctrl (FC) Antibody	BioLegend	Cat# 400121 (also 400122), RRID:AB_326443
APC Mouse IgG2a, κ Isotype Control	BD Biosciences	Cat# 555576, RRID:AB_398604
**Bacterial and virus strains**		
Mycobacterium tuberculosis H37Rv	Douglas Young lab	N/A
Mycobacterium tuberculosis H37Rv-pTEC15	Max Gutierrez lab	unpublished
Mycobacterium tuberculosis H37Rv-pTEC19	Max Gutierrez lab	PMID: 32938685
**Chemicals, peptides, and recombinant proteins**		
Essential 8™ Medium	Thermo Fisher Scientific	Cat# A1517001

mTeSR Plus	STEMCELL Technologies	Cat# 100-0276
Y-27632	Tocris Bioscience	Cat# 1254
Vitronectin XF	STEMCELL Technologies	Cat# 07180
Corning® Matrigel® Growth Factor Reduced (GFR) Basement Membrane Matrix	Merck	Cat# 356230
Corning® Matrigel® Basement Membrane Matrix	Merck	Cat# 356234
DPBS, no calcium, no magnesium	Thermo Fisher Scientific	Cat# 14190144
Versene solution	Thermo Fisher Scientific	Cat# 15040066
Gentle Cell Dissociation Reagent	STEMCELL Technologies	Cat# 100-0485
TrypLE™ Express Enzyme (1X), no phenol red	Thermo Fisher Scientific	Cat# 12563011
STEMdiff™ Definitive Endoderm Kit	STEMCELL Technologies	Cat# 05110
IMDM	Thermo Fisher Scientific	Cat# 12440061
Corning™ cellgro™ DMEM/Ham's Medium F-12 Mix	Thermo Fisher Scientific	Cat# 15363541
N-2 Supplement (100X)	Thermo Fisher Scientific	Cat# 17502048
B-27™ Supplement (50X), minus vitamin A	Thermo Fisher Scientific	Cat# 12587010
Bovine Albumin Fraction V (7.5% solution)	Thermo Fisher Scientific	Cat# 15260037
1-Thioglycerol	Merck	Cat# M6145
L-Ascorbic acid	Merck	Cat# A4544
GlutaMAX™ Supplement	Thermo Fisher Scientific	Cat# 35050038
Primocin®	InviviGen	Cat# ant-pm-05
SB431542	Selleck Chemicals	Cat# S1067-SEL-10mg
Dorsomorphin	Selleck Chemicals	Cat# S7840-SEL-5mg
Recombinant Human BMP-4	Peprotech	Cat# 120-05
CHIR99021	Selleck Chemicals	Cat# S1263
Retinoic acid	Merck	Cat# R2625
Recombinant Human KGF/FGF-7 Protein	R&D Systems, Inc.	Cat# 251-KG
DAPT	Merck	Cat# D5942
Recombinant Human FGF-10	Peprotech	Cat# 100-26
Dexamethasone	Merck	Cat# D4902
8-Bromoadenosine 3’,5’-cyclic monophosphate	Merck	Cat# B7880
3-Isobutyl-1-methylxanthine	Merck	Cat# I5879
AggreWell™800	STEMCELL Technologies	Cat# 34815
Anti-Adherence Rinsing Solution	STEMCELL Technologies	Cat# 07010
Recombinant Human VEGF165	Peprotech	Cat# 100-20
Recombinant Human SCF	Peprotech	Cat# 300-07
TheraPEAK™ X-VIVO™-15 Serum-free Hematopoietic Cell Medium	Lonza	Cat# BEBP02-061Q
2-Mercaptoethanol	Thermo Fisher Scientific	Cat# 21985023
Recombinant Human M-CSF	Peprotech	Cat# 300-25
Recombinant Human IL-3	Peprotech	**Cat# 200-03**
Recombinant Human GM-CSF	Peprotech	Cat# 300-03
DMEM/F-12, HEPES	Thermo Fisher Scientific	Cat# 11330057
Neurobasal™ Medium	Thermo Fisher Scientific	Cat# 21103049

StemPro™-34 SFM (1X)	Thermo Fisher Scientific	Cat# 10639-011
Forskolin, Adenylyl cyclase activator	Abcam	Cat# ab120058
Paraformaldehyde 16% Aqueous Solution EM Grade	Electron Microscopy Sciences	Cat# 15710
Saponin Quillaja sp.	Merck	Cat# S4521
Bovine Serum Albumin	Cell Signaling Technology	Cat# 9998
DAPI (4',6-Diamidino-2-Phenylindole, Dihydrochloride)	Thermo Fisher Scientific	Cat# D1306
Fluorescence Mounting Medium (Dako Omnis)	Agilent	Cat# GM30411-2
Intracellular Staining Permeabilization Wash Buffer (10X)	BioLegend	Cat# 421002
DMEM, high glucose, pyruvate	Thermo Fisher Scientific	Cat# 11995073
Fetal Bovine Serum, value	Thermo Fisher Scientific	Cat# A5256701
Ethanol, 99.8%, for HPLC, absolute	Thermo Fisher Scientific	Cat# 12337163
ACCUTASE™	STEMCELL Technologies	Cat# 07920
Glutaraldehyde solution	Merck	Cat# G5882
HEPES solution	Merck	Cat# H0887
OSMIUM TETROXIDE 4% SOL.-100ML	TAAB	Cat# O011
POTASSIUM FERRICYANIDE-100G	TAAB	Cat# P018
Thiocarbohydrazide	Merck	Cat# 223220
Uranyl acetate dihydrate	Agar scientific	Cat# AGR1260A
Durcupan™ ACM	Merck	Cat# 44610
OCT Compound	Agar Scientific	Cat# AGR1180
BD DIFCO™ Middlebrook 7H9 Broth 500g	BD Biosciences	Cat# 271310
CD BBL™ Middlebrook ADC Enrichment	BD Biosciences	Cat# 212352
Glycerol	Merck	Cat# G9012
TWEEN® 80	Merck	Cat# P1754
Hygromycin B (50 mg/mL)	Thermo Fisher Scientific	Cat# 10687010
**Critical commercial assays**		
CD144 (VE-Cadherin) MicroBeads, human	Miltenyi Biotec	Cat# 130-097-857
Alexa Fluor™ 647 Phalloidin	Thermo Fisher Scientific	Cat# A22287
eBioscience™ Calcein Blue AM Viability Dye	Thermo Fisher Scientific	Cat# 65-0855-39
Low Density Lipoprotein from Human Plasma, Acetylated, Alexa Fluor™ 594 Conjugate	Thermo Fisher Scientific	Cat# L35353
Taqman hs *NKX2-1* probe	Thermo Fisher Scientific	Cat# 4331182, Assay ID: Hs00968940_m1
Taqman hs *SFTPC* probe	Thermo Fisher Scientific	Cat #4351372 Assay ID: Hs07292706_g1
Taqman hs *SLC34A2* probe	Thermo Fisher Scientific	Cat# 4331182 Assay ID: Hs00197519_m1
Taqman hs *PDPN* probe	Thermo Fisher Scientific	Cat# 4331182 Assay ID: Hs00366766_m1
Taqman hs *CAV1* probe	Thermo Fisher Scientific	Cat# 4331182 Assay ID: Hs00971716_m1
Taqman hs *CD31* probe	Thermo Fisher Scientific	Cat# 4331182 Assay ID: Hs01065279_m1
Taqman hs *CD144* probe	Thermo Fisher Scientific	Cat# 4331182 Assay ID: Hs00901465_m1

Taqman hs *CD34* probe	Thermo Fisher Scientific	Cat# 4331182 Assay ID: Hs02576480_m1
Taqman hs *vWF* probe	Thermo Fisher Scientific	Cat# 4331182 Assay ID: Hs01109446_m1
RNeasy Mini Kit	QIAGEN	Cat# 74104
QuantiTect Reverse Transcription Kit	QIAGEN	Cat# 205311
TaqMan™ Universal PCR Master Mix	Thermo Fisher Scientific	Cat# 4304437
Chromium Next GEM Single Cell 3′ Kit v3.1	10X Genomics	Cat# CG000315
Bio-Plex Pro Human Cytokine Screening Panel, 48-Plex	BioRad	Cat# 12007283
RNAscope™ LS Multiplex Fluorescent assay	Bio-Techne	Cat# 322800
RNAscope hs *IL6* probe	Bio-Techne	Cat# 310378-C4
RNAscope hs *IL8* probe	Bio-Techne	Cat# 310388-C3
Opal 570 Reagent Pack	Akoya Biosciences	Cat# FP1488001KT
Opal 780 Reagent Pack	Akoya Biosciences	Cat# FP1501001KT
LIVE/DEAD™ Fixable Orange (602) Viability Kit, for 561 nm excitation	Thermo Fisher Scientific	Cat# L34983
**Deposited data**		
scRNA-seq data	GEO	GSE252601
**Experimental models: Cell lines**		
KOLF2HPSI0114i-kolf_2 human iPSC	Public Health England Culture Collections	Cat# 77650100
KOLF2HPSI0114i-kolf_2 human iPSC *ATG14KO*	Max Gutierrez lab	PMID: 36959508
**Software and algorithms**		
Prism v10.0.1	GraphPad	RRID:SCR_002798
FlowJo v10.8.1	FlowJo, LLC	RRID:SCR_008520
Fiji v2.9.0/1.54f	NIH	RRID:SCR_002285
QuPath v0.4.4	QuPath	RRID:SCR_018257
**Other**		
AX12 organ-on-chip	AlveoliX	N/A
AXExchanger	AlveoliX	N/A
^{AX}Breather	AlveoliX	N/A
LS Columns	Miltenyi Biosciences	Cat# 130-042-401
MACS Multi-stand	Miltenyi Biosciences	Cat# 130-042-303
autoMACS® Rinsing Solution	Miltenyi Biosciences	Cat# 130-091-222
Epithelial Volt/Ohm Meter 2	World Precision Instruments	Cat# EVOM2
Epithelial Volt/Ohm Meter 3	World Precision Instruments	Cat# EVOM3
STX100 Electrode Corning 96	World Precision Instruments	Cat# STX100C96
EVOM3 legacy probe kit	World Precision Instruments	Cat# 99672
PELCO BioWave® Pro+ Microwave Processing System	Pelco	Cat# 36700-230
Leica EM CPD300 Critical Point Dryer	Leica microsystems	N/A

DiATOME 35° Ultra - Diamond Knives	DiATIME	Cat# AGG339
Leica UC7 Ultramicrotome	Leica microsystems	N/A
JEOL1400FLASH	JOEL	N/A
Olympus CSU-W1 SoRa Spinning Disk Microscope	Olympus	N/A
STELLARIS 8 Confocal Microscope Platform	Leica microsystems	N/A
BD LSRFortessa™ Cell Analyzer	BD Biosciences	Cat# 647800
BD FACSAria™ Fusion Flow Cytometer	BD Biosciences	Cat# 658282
SP Bel-Art Flowmi 40 Micron Cell Strainers for 1000 Microliter Pipette Tips	Bel-Art	Cat# H13680-0040
LUNA-FX7™ Automated Cell Counter	Logos Biosystems	Cat# L70002
Acridine Orange/Propidium Iodide Stain	Logos Biosystems	Cat# F23001
Agilent TapeStation	Agilent	N/A
NovaSeq 6000 Sequencing System	Illumina	N/A
Corning™ Costar™ Spin-X™ Centrifuge Tube Filters, CA Membrane	Thermo Fisher Scientific	Cat# 8160
Bio-Plex® 200 System	BioRad	Cat# 171000201
CM3050S Cryostat	Leica microsystems	N/A
BOND RX Fully Automated Research Stainer	Leica microsystems	N/A
Vectra Polaris™ Automated Quantitative Pathology Imaging System	Akoya Biosciences	N/A

Data S1. (separate file)

Results from differential expression analysis in each pairwise comparison.
